# An Injectable Biopolymer Hydrogel Complex (PN/HA/B3) for Facial Skin Redensification and General Rejuvenation: Clinical Report on Device Safety and Efficacy

**DOI:** 10.3390/jfb17050254

**Published:** 2026-05-20

**Authors:** Alexandre Porcello, Kelly Lourenço, Cíntia Marques, Wassim Raffoul, Marco Cerrano, Lee Ann Applegate, Alexis E. Laurent

**Affiliations:** 1 Development Department, LOUNA REGENERATIVE SA, CH-1228 Plan-les-Ouates, Switzerland; a.porcello@louna-aesthetics.com (A.P.); k.lourenco@louna-aesthetics.com (K.L.); c.marques@louna-aesthetics.com (C.M.); 2 Plastic and Reconstructive Surgery, Ensemble Hospitalier de la Côte, CH-1110 Morges, Switzerland; wassim.raffoul@ehc.vd.ch; 3 Entourage Aesthetic Clinic, CH-1003 Lausanne, Switzerland; m.cerrano@entourage.ch; 4 Regenerative Therapy Unit, Lausanne University Hospital, University of Lausanne, CH-1066 Epalinges, Switzerland; lee.laurent-applegate@unil.ch; 5 Center for Applied Biotechnology and Molecular Medicine, University of Zurich, CH-8057 Zurich, Switzerland; 6 Oxford OSCAR Suzhou Center, Oxford University, Suzhou 215123, China; 7 Manufacturing Department, LAM Biotechnologies SA, CH-1066 Epalinges, Switzerland; 8 Manufacturing Department, TEC-PHARMA SA, CH-1038 Bercher, Switzerland

**Keywords:** Cutometer, Dermascan, GAIS (Global Aesthetic Improvement Scale), hyaluronic acid, niacinamide, polynucleotides, skin elasticity, skin hydration, skin quality, skin texture

## Abstract

This study evaluated the safety and effectiveness of HYDRAGEL A2, an injectable medical device containing hyaluronic acid (HA), polynucleotides (PN), and niacinamide, for improving facial skin quality. These ingredients are increasingly recognized for their synergistic effects in aesthetic medicine, with HA and PN providing hydration and skin support, and niacinamide offering anti-inflammatory and antioxidant properties. A prospective, open-label clinical investigation was conducted on 42 female subjects (mean age 45 ± 1 years, Fitzpatrick skin phototypes II-V) to assess skin elasticity, hydration, and mild skin depression correction following cheek area injections. Efficacy was measured using the Global Aesthetic Improvement Scale (GAIS), Antera 3D^®^ (texture), Cutometer^®^ (elasticity/firmness), Corneometer^®^ (hydration), and Dermascan^®^ (density/thickness) devices at baseline (D0), week 2 (W2/D14), and week 6 (W6/D42). GAIS values showed significant overall facial improvement (*p* < 0.001) by both investigators and subjects, where 100% of subjects rated their appearance as improved immediately post-injection (D0), with sustained improvements at D42. Objective measurements revealed significant improvements in skin texture (reduced roughness), elasticity, firmness, hydration (*p* < 0.001), density, and thickness, demonstrating the combined benefits of the HA, PN, and niacinamide blend. Injection site reactions, primarily mild and transient, were reported immediately post-injection. Investigators and subjects reported high satisfaction with the product’s ease of use and aesthetic outcomes. Globally, HYDRAGEL A2, leveraging the established benefits of HA, PN, and niacinamide, was well-tolerated and effectively enhanced facial skin quality, demonstrating significant and sustained improvements in monitored skin parameters. The study concludes that this combination of ingredients, formulated in HYDRAGEL A2, provides a well-tolerated approach associated with improvements in skin quality.

## 1. Introduction

Facial skin aging, a complex and dynamic process, is orchestrated by a confluence of intrinsic genetic predispositions, inherent biological transformations, and cumulative extrinsic environmental insults [1,2]. This intricate interplay manifests as discernible alterations in skin quality, profoundly impacting aesthetic perception and, consequently, psychological well-being [3]. Hallmark indicators of this aging cascade include textural irregularities, diminished elasticity, compromised hydration, and reduced dermal density [4,5,6]. These changes transcend mere superficiality, reflecting underlying physiological shifts such as the age-related decline in biomechanical properties like skin elasticity that progressively erode the skin’s structural integrity and functional competence [7]. Consequently, there is an increasing demand for scientifically validated interventions that target cellular and extracellular matrix (ECM) alterations underlying skin ageing. These include both invasive and noninvasive facial rejuvenation procedures, characterized by trends toward soft-tissue fillers, platelet-rich plasma, lasers, and other energy-based modalities aimed at wrinkle reduction and youth restoration. Overall, the ultimate goal of these approaches consists in restoring dermal soft-tissue architecture, improving biomechanical properties, and re-establishing a more youthful phenotype [8,9]. In contemporary clinical practice, this goal has driven a profound paradigm shift from the purely volumetric ‘filling’ of static rhytides to the holistic, two-dimensional enhancement of global skin quality. While traditional crosslinked dermal fillers excel at replacing deep structural volume loss, they are often inappropriate for superficial intradermal injection due to their high viscosity and the risk of the Tyndall effect or surface irregularities. Consequently, there is a critical clinical demand for highly spreadable, non-crosslinked biostimulatory scaffolds that can be safely dispersed across broad aesthetic units—such as the superficial cheek and perioral zones—to achieve true tissue redensification and functional rejuvenation without artificially altering the patient’s natural facial contours.

Hyaluronic acid (HA), used both topically and through intradermal or subcutaneous injections, has demonstrated proven efficacy in improving skin quality and inducing dermal remodeling, as supported by various clinical studies [10,11]. While crosslinked HA tends to deliver more sustained and long-term results, linear HA used as a monotherapy has also shown significant clinical benefits, primarily in enhancing cutaneous hydration. However, for other markers of skin quality (e.g., firmness, texture, and radiance) the clinical outcomes with linear HA appear more inconsistent [11,12,13]. Notably, a 2018 consensus identified crosslinked HA-based skin boosters as the preferred first-line approach for skin hydration treatments [14]. Notwithstanding, HA effects may not fully address the multifactorial nature of skin aging [13,14,15]. The visible signs of aging are not isolated phenomena, but rather the culmination of complex interactions between diverse skin properties, necessitating a holistic and multimodal strategy [13,16]. From a macromolecular perspective, the clinical utility of linear HA as a standalone biostimulator is inherently limited by its rapid biological turnover [17]. Endogenous linear HA is highly susceptible to swift enzymatic cleavage by local hyaluronidases, as well as rapid depolymerization by reactive oxygen species (ROS) generated by environmental stressors [17]. To overcome these pharmacokinetic limitations without resorting to chemical crosslinking—which can alter the polymer’s native biological signaling—modern formulation strategies must employ synergistic bioactive molecules.

In recent years, dermatological research has increasingly focused on injectable strategies to improve skin quality [13,18,19,20,21]. Among the most extensively investigated candidates in this regard are polynucleotides (PN) and niacinamide, both of which have demonstrated distinct and complementary mechanisms of action in skin rejuvenation [13,20,21,22,23,24,25,26]. Polynucleotides (PN), high-molecular-weight biopolymers derived from purified DNA fragments, have been increasingly incorporated into injectable hydrogel formulations in aesthetic medicine. Within the hydrogel matrix, PN chains contribute to the structural and rheological properties of the complex, enhancing its viscosity, and scaffolding capacity within the dermal compartment. Upon gradual biodegradation of the hydrogel in situ, the released PN fragments and nucleotide monomers become available as building blocks within the local tissue environment, where they may support physiological tissue maintenance processes, including ECM homeostasis [20,22,27]. Niacinamide (i.e., vitamin B3), a well-characterized hydrosoluble compound, is incorporated into formulations as a stabilizing and protective excipient. Its established antioxidant properties contribute to preserving the structural integrity of the HA polymer network against oxidative depolymerization caused by ROS generated by environmental stressors such as ultraviolet (UV) radiation. By mitigating ROS-mediated degradation of the hydrogel matrix, niacinamide serves a primarily protective function, extending the functional residence time of the HA scaffold within the injected tissue [23,25,28].

The combination of HA, PN, and niacinamide in HYDRAGEL A2 is designed to sequentially address multiple facets of skin aging, providing a holistic and comprehensive approach to skin rejuvenation. By delivering these compounds directly into the dermal layers, HYDRAGEL A2 aims to achieve enhanced efficacy and sustained improvements in skin quality. The scientific rationale behind this formulation is based on the postulate that targeted and combined delivery of these agents can yield profound and long-lasting enhancements in key facial skin attributes.

This clinical investigation evaluates the safety and effectiveness of the HYDRAGEL A2 device in improving skin quality, including texture, elasticity, hydration, density, and thickness. The novelty of this study lies in the acquisition of human clinical data on an injectable device formulation combining these three powerful ingredients. Specifically, this trial evaluates the safety and effectiveness of HYDRAGEL A2 in enhancing facial skin aesthetics. The anticipated findings will provide valuable insights into the efficacy, safety, and practical applications of HYDRAGEL A2, contributing to advancements in facial skin aesthetics and the management of visible signs of aging. The primary objective is to determine the benefit/risk profile of HYDRAGEL A2 in the intended target population. Specific sub-objectives include evaluating its efficacy in improving facial skin quality—encompassing skin texture, firmness/elasticity, hydration, thickness/density—at day 14 (i.e., week 2 (W2)) and at day 42 (i.e., week 6 (W6)) post-injection and assessing its safety and tolerance throughout the study period. This research aims to contribute significantly to the growing body of scientific and clinical knowledge surrounding injectable skin rejuvenation therapies, providing evidence-based insights into the potential of HA, PN, and niacinamide in enhancing skin quality and addressing the multifaceted challenges of facial aging.

## 2. Materials and Methods

### 2.1. Clinical Study Design

This clinical trial was registered in a publicly accessible database under registration number ISRCTN37981524. The study received ethics approval on 17 January 2024 from the Clinical Research Regulatory Council (Atchia Building, Suffren Street, Port-Louis 11405, Mauritius), under reference number 2223CMPH145. This study was designed as a prospective, open-label, single-center clinical trial, conducted at a contract research organization (CRO; CIDP Ltée, Phoenix, Mauritius), to systematically evaluate the safety and efficacy of HYDRAGEL A2 for improving facial skin quality. This trial aimed to comprehensively assess the product’s ability to enhance facial skin quality, specifically targeting skin elasticity, hydration, and radiance, and to correct mild skin depressions through dermal filling and redensification. Furthermore, the study sought to determine the product’s tolerability and to delineate its impact on facial skin radiance, thereby providing a robust assessment of its clinical utility. Participants served as their own baseline controls, allowing for a direct comparison of pre- and post-injection skin parameters, minimizing inter-subject variability.

The clinical trial was conducted in strict adherence to Good Clinical Practice (GCP) guidelines and the ethical principles outlined in the Declaration of Helsinki [29]. Prior to the commencement of the study, the clinical protocol received approval from an Independent Ethics Committee, and written informed consent was obtained from each participant, ensuring their voluntary participation and explicit consent for the publication of the study results.

The primary efficacy endpoint was the investigator’s Global Aesthetic Improvement Scale (GAIS) evaluation at two weeks (Day 14) post-injection, providing a standardized assessment of aesthetic improvement. Secondary endpoints included objective and quantitative measurements of skin texture (roughness) using the Antera 3D^®^ device, skin firmness/elasticity (Cutometer^®^), skin hydration (Corneometer^®^), and skin density/thickness (Dermascan^®^) at both Day 14 and Day 42. These objective measurements provided a comprehensive evaluation of the product’s impact on various skin parameters. Additionally, the study assessed the proportion of subjects demonstrating improvements via independent investigator GAIS assessment at Day 42, injector satisfaction with the injection process immediately post-injection (Day 0), photographic documentation of intervention effects using standardized 2D photography, safety assessment through injection site reaction (ISR) questionnaires filled out daily by subjects and at visits by investigators, and subject satisfaction with global aesthetic improvement and intervention outcomes at Day 14 and Day 42.

The primary performance measure was the responder rate based on the GAIS, with a responder defined as a subject exhibiting “very much improved”, “much improved”, or “improved” scores. The study hypothesized that at least 65% of subjects would be responders, aiming to reject the null hypothesis of less than 40% responders, with a power of 90% and an alpha of 5%, using a two-sided exact one-sample binomial test. This statistical approach ensured an assessment of the product’s efficacy.

Participants underwent a screening visit (Day-30) to ensure eligibility, followed by baseline assessments (Day-3 to Day 0), where skin parameters were meticulously measured using the aforementioned devices. At baseline (Day 0), HYDRAGEL A2 was injected into both cheeks by a highly trained investigator, with a maximum total volume of 3.6 mL (i.e., 1.8 mL per cheek), adhering to standardized injection techniques to minimize variability. Injector satisfaction with the injection process was immediately recorded using a detailed subjective evaluation questionnaire, assessing factors such as ease of injection, product flow, and patient comfort. Follow-up evaluations were performed at Day 14 and Day 42, including objective skin measurements using the Antera 3D^®^, Cutometer^®^, Corneometer^®^, and Dermascan^®^ devices, and GAIS assessments by both the principal investigator and an independent investigator at Day 42. Standardized 2D photographs were taken at each patient visit, using consistent lighting and positioning, to visually document intervention effects and facilitate comparative analysis. Subjects diligently completed daily ISR logs throughout the study duration, providing a comprehensive record of any adverse events. Investigators conducted thorough ISR assessments at Day 0, Day 14, and Day 42, ensuring a comprehensive safety evaluation. Subject satisfaction with intervention outcomes was recorded using detailed questionnaires at Day 14 and Day 42, providing valuable insights into patient-reported outcomes. The total study duration was four months, with each subject participating for six weeks. Investigators underwent rigorous training on all clinical scales, ensuring consistency and minimizing inter-rater variability and bias. Forty-four female subjects, aged 30 to 59, were enrolled to ensure inclusion of at least 40 evaluable subjects, accounting for potential dropouts, with two subjects withdrawing consent. Study completion was defined as the ultimate subject’s last visit (Day 42).

### 2.2. Demographics of the Study Population

A total of 44 subjects were enrolled in this prospective clinical trial. Of these, 43 received the HYDRAGEL A2 injections, and 42 completed the study protocol. The participant cohort comprised exclusively female subjects, with ages ranging from 30 to 59 years, and a mean age of 45 ± 1 years (Table 1). To ensure a representative sample, subjects with Fitzpatrick skin phototypes II through V were included, reflecting a diverse range of skin pigmentation. Specifically, the study included 1 subject with phototype II, 12 subjects with phototype III, 17 subjects with phototype IV, and 13 subjects with phototype V.

Inclusion criteria were not designed to exclude subjects based on ethnicity, thereby ensuring a heterogeneous population. All participants were required to be in a generally healthy state, with normal skin conditions, to minimize confounding variables and ensure that observed changes could be attributed primarily to the investigational device. This stringent inclusion criterion aimed to provide a focused assessment of HYDRAGEL A2’s efficacy and safety within a well-defined cohort.

### 2.3. Study Workflow

The study protocol comprised five distinct successive visits for each participant, designed to comprehensively evaluate the safety and efficacy of HYDRAGEL A2. These included a screening visit (Day-30), a baseline visit (Day-3 to Day 0), the injection visit (Day 0), and two follow-up visits at Week 2 (Day 14) and Week 6 (Day 42). During the initial screening visit (Day-30), participants underwent a thorough general evaluation to confirm their eligibility for the study. This included the provision and signing of informed consent, a detailed verification of inclusion and exclusion criteria to ensure participant safety and study integrity, and a comprehensive collection of medical history to identify any potential confounding factors. The baseline visit, conducted between Day-3 and Day 0, established a comprehensive pre-treatment profile for each participant.

At the injection visit (Day 0), a comprehensive safety evaluation was conducted, including the recording of any adverse events and concomitant medications. The investigator then assessed the participant’s skin condition using the Global Aesthetic Improvement Scale (GAIS) to establish a baseline aesthetic assessment. Subsequently, HYDRAGEL A2 was administered via injection into both cheeks, with a maximum total volume of 3.6 mL (i.e., 1.8 mL per cheek), adhering to standardized injection protocols. Immediate injection site reactions (ISRs) were meticulously documented by the investigator, and participants were provided with detailed daily ISR logs to record any post-injection symptoms. Participants were provided clear post-administration instructions, including avoidance of sun exposure, extreme temperatures, and cosmetic products for 12 h post-injection, to minimize potential confounding factors and ensure optimal intervention outcomes.

Follow-up visits at Week 2 (Day 14) and Week 6 (Day 42) were crucial for assessing both the efficacy and safety profiles of HYDRAGEL A2. These visits included: recording of any adverse events and concomitant medications; GAIS assessment to evaluate aesthetic improvement; verification of subject-reported ISRs; investigator assessment of ISRs; standardized 2D facial photography to document visual changes; and repeat measurements of skin texture, elasticity, density, thickness, and hydration using the same instruments as at the baseline. These follow-up assessments allowed for a longitudinal evaluation of treatment effects and safety. Throughout the study, from the initial screening visit to the final visit at Week 6, all adverse events and concomitant medications were meticulously tracked and documented, ensuring a comprehensive safety profile. A detailed study flowchart, outlining the sequence of events and assessments, is presented in Table 2.

### 2.4. Patient Selection Methodology

This single-center, prospective, open-label study enrolled 44 female subjects, aiming to achieve a minimum of 40 evaluable participants to ensure adequate statistical power. The participant selection process was designed to recruit a homogeneous cohort while reflecting a diverse range of skin phototypes. Subjects were eligible if they were female, aged 30 to 60 years, of any ethnicity, and presented with Fitzpatrick skin phototypes II-V, indicating a range from light to dark skin pigmentation.

Specific inclusion criteria were meticulously defined to ensure a well-characterized study population. These criteria mandated that participants: (1) be female, to maintain homogeneity in hormonal influences on skin quality; (2) be aged between 30 and 60 years, targeting a demographic experiencing noticeable skin aging; (3) be of any ethnicity, to broaden the applicability of the study results; (4) have Fitzpatrick skin phototypes II to V, encompassing a wide range of skin pigmentation; (5) seek improvement of their facial skin quality, ensuring a clear clinical indication; (6) consent to photographic documentation, essential for objective assessment; (7) agree to abstain from other facial aesthetic procedures during the study, to isolate the effects of HYDRAGEL A2; (8) be in good general and mental health, minimizing confounding variables; (9) be able to read, understand, and provide written informed consent, ensuring ethical compliance; (10) agree to cooperate with study procedures, ensuring adherence to the protocol; (11) be deemed compliant by the investigator, ensuring data integrity; and (12) be capable of following study rules and schedules, minimizing protocol deviations.

Exclusion criteria were rigorously defined to minimize confounding factors and ensure subject safety. Participants were excluded if they: (1) had any systemic disorder or skin disease that could interfere with study results, ensuring that observed changes were attributable to the investigational device; (2) had a history of severe allergies or anaphylactic shock, mitigating the risk of severe adverse events; (3) had known hypersensitivity to HYDRAGEL A2 components, ensuring product safety; (4) suffered from autoimmune diseases, which could affect skin responses; (5) had cutaneous disorders, inflammation, or an infection at the intervention site, preventing accurate assessment of intervention effects; (6) had a medical history suggesting sensitivity to the device, minimizing potential adverse reactions; (7) had bleeding disorders or were undergoing treatment with thrombolytics or anticoagulants, mitigating bleeding risks; (8) had a tendency to form keloids, hypertrophic scars, or other skin healing disorders, ensuring predictable healing outcomes; (9) were currently undergoing skin treatments, isolating the effects of HYDRAGEL A2; (10) were pregnant, breastfeeding, or planning pregnancy during the study, avoiding potential fetal risks; (11) had a positive urine pregnancy test at baseline, ensuring non-pregnancy status; (12) were under legal guardianship, ensuring informed consent; (13) could not be contacted by telephone in case of emergency, ensuring participant safety; (14) were participating in another biomedical research study or in a wash-out period from a previous study, preventing confounding variables; or (15) had intellectual or mental incapacitation, ensuring informed consent and protocol adherence.

Data for withdrawn subjects were meticulously recorded in the electronic case report form (eCRF) and source documents, and follow-up was conducted to determine reasons for withdrawal and any associated adverse events, ensuring comprehensive safety monitoring. Major protocol deviations, including prohibited treatments, missed injections, and missed study visits, were meticulously documented. Participants received financial compensation for their participation, with full compensation provided for adverse event-related withdrawals, ensuring fair compensation and minimizing attrition.

### 2.5. Investigational Test Item

HYDRAGEL A2, classified as a Class III medical device under Regulation (EU) 2017/745, Rule 8, Chapter III, is presented as a sterile, translucent, and resorbable hydrogel specifically formulated for intradermal injection. The product is packaged in 2 mL pre-filled, single-use glass syringes. The hydrogel’s composition is designed to achieve optimal skin rejuvenation through a synergistic combination of key components:

- Hyaluronic Acid (HA): At a concentration of 7.5 mg/mL and a MW of 1.8 MDa, derived from biofermentation, forms the core of the formulation.
- Polynucleotides (PN): At a concentration of 11.25 mg/mL and a MW of 1.5 MDa.
- Niacinamide: At a concentration of 15 mg/mL.

A maximum volume of 3.6 mL is administered per subject (i.e., 1.8 mL per cheek) at baseline (Day 0), with the exact volume determined by the injector based on individual patient needs and anatomical considerations (the mean volume was 1.4 mL per cheek). This personalized approach ensures optimal aesthetic outcomes and minimizes the risk of overcorrection.

Contraindications for HYDRAGEL A2 include known hypersensitivity to any component, a history of severe allergies or anaphylactic shock, autoimmune diseases, cutaneous disorders at the injection site, bleeding disorders, keloid formation tendencies, pregnancy, breastfeeding, and concurrent skin treatments. These contraindications are essential for ensuring patient safety and minimizing the risk of adverse events.

### 2.6. Administration Protocol and Injection Technique

HYDRAGEL A2 was administered exclusively by authorized healthcare professionals, meticulously selected for their comprehensive understanding of facial anatomy and extensive experience in similar intradermal injection procedures. This stringent requirement ensured that the product was administered with the highest level of precision and safety, adhering to local regulatory guidelines.

The injection procedure commenced with meticulous disinfection of the targeted injection site using an appropriate antiseptic solution, such as chlorhexidine or povidone-iodine, to minimize the risk of bacterial contamination and subsequent infection. Following this, the healthcare professional carefully removed the tip-cap from the pre-filled HYDRAGEL A2 syringe, ensuring aseptic handling. A sterile cannula (i.e., 25 G ×50 mm, 2-inch STERiGLIDE^TM^), selected based on the injector’s professional judgment and the specific anatomical region being injected, was then securely screwed onto the syringe. The HYDRAGEL A2 gel was administered slowly and evenly into the dermis or subcutaneous tissue, employing a technique that allowed for precise placement and optimal distribution of the product. The injection sites were specifically localized within the cheek areas, as illustrated in Figure S1, to achieve the desired aesthetic improvements.

Throughout the administration, strict adherence to aseptic techniques and standard medical practices was maintained to minimize the risk of complications. The healthcare professional exercised caution to avoid intravascular injection, which could lead to serious adverse events, muscle injection, which could result in discomfort and suboptimal product distribution, and injection into areas previously treated with non-resorbed products or permanent implants, which could lead to unpredictable interactions.

Patients receiving antiplatelet medications, such as aspirin or clopidogrel, were informed of the increased risk of hematomas and bleeding during the injection procedure. This informed consent process allowed patients to make informed decisions regarding their treatment. If a touch-up intervention was deemed necessary to achieve optimal correction, it was scheduled at least two weeks post-initial injection, allowing for the resolution of any potential side effects and accurate assessment of the intervention’s initial effects. Patients were advised to avoid prolonged sun exposure, extreme temperatures (i.e., below 0 °C), and sauna/hammam sessions for two weeks following the intervention, as these factors could exacerbate local swelling and inflammation. Additionally, they were instructed to refrain from using cosmetic products for 12 h post-injection, minimizing the risk of irritation and infection.

During the study, Diaseptyl^®^ (chlorhexidine gluconate; Laboratoires Pierre Fabre, Boulogne, France) was used for skin disinfection, providing a broad spectrum of antimicrobial activity. Emla^®^ cream (lidocaine/prilocaine; Aspen Pharma Trading Limited, Dublin, Ireland) was made available for topical anesthesia upon patient request, enhancing patient comfort during the procedure. The administration protocol was meticulously followed to ensure the safety and efficacy of HYDRAGEL A2 in improving facial skin quality.

### 2.7. Outcome Endpoints

Clinical monitoring was conducted to ensure that the rights and well-being of human subjects were protected, that the conduct of the trial was in compliance with the approved protocol, with ICH GCP Guidelines, and with applicable regulatory requirements and that the reported trial data were accurate, complete, and verifiable.

#### 2.7.1. Efficacy Endpoints

Efficacy was primarily determined by the proportion of subjects demonstrating a clinically meaningful improvement in the injected area, as assessed by the investigator using the Global Aesthetic Improvement Scale (GAIS) at 2 weeks (Day 14) post-injection. The GAIS, a validated subjective rating scale, allowed investigators to grade improvement relative to baseline, with “very much improved”, “much improved”, or “improved” scores defining a positive treatment response, reflecting a clinically major aesthetic enhancement. This subjective assessment provided a critical measure of the product’s perceived effectiveness from the investigator’s perspective.

Secondary efficacy endpoints were designed to provide a comprehensive understanding of HYDRAGEL A2’s effects on various aspects of skin quality. Objective measurements were conducted at 2 weeks (Day 14) and 6 weeks (Day 42) post-injection, focusing on predefined cheek zones (Z1 and Z2) to minimize variability in measurement acquisition and ensure consistency across subjects. These predefined zones were anatomically standardized to allow for precise comparisons of pre- and post-treatment skin parameters.

Skin texture (roughness) was assessed using the Antera 3D^®^ handheld camera, a sophisticated imaging system that captured and analyzed skin surface topography with high resolution. This device provided quantitative data on skin roughness parameters, allowing for objective evaluation of textural improvements. Skin firmness/elasticity was quantified using the Cutometer^®^ dual MPA 580, a device that applied negative pressure to measure skin deformation and recovery, yielding parameters such as extensibility (Ue), immediate retraction (Ur), and overall elasticity (Ur/Uf). These biomechanical parameters provided objective measures of the skin’s viscoelastic properties. Skin hydration was evaluated using the Corneometer^®^ CM 825, which measured the dielectric constant of the stratum corneum, reflecting water content and providing a quantitative assessment of skin hydration levels. Skin density and thickness were assessed using the Dermascan^®^ C USB ultrasound system, a high-resolution imaging tool that provided detailed cross-sectional images of skin structures (i.e., epidermis, dermis, hypodermis), allowing for objective evaluation of dermal thickness and density changes.

All objective measurements were performed by the same trained technician, adhering to standardized operating procedures, to ensure consistency and minimize inter-operator variability. Any deviations from the standard protocol were meticulously documented in the eCRF, ensuring transparency and data integrity.

Additional efficacy assessments included the proportion of subjects showing improvement, as evaluated by an independent investigator using the GAIS at 6 weeks (Day 42), providing an unbiased assessment of long-term aesthetic outcomes. Injector satisfaction with injection quality was assessed via a subjective questionnaire on Day 0, evaluating injection, and immediate results, providing insights into the product’s handling characteristics. Visual documentation of treatment effects was captured through standardized 2D photography using a VISIA-CR^®^ (Canfield Scientific, Parsippany, NJ, USA) a high-resolution imaging system that captured standardized facial images under controlled lighting conditions, allowing for objective visual comparisons of pre- and post-intervention facial features. Subject satisfaction with global aesthetic improvement and treatment outcomes was measured through detailed questionnaires at 2 weeks (Day 14) and 6 weeks (Day 42) post-injection, assessing improvements in skin firmness, elasticity, hydration, and overall satisfaction, providing valuable patient-reported outcomes.

#### 2.7.2. Safety and Tolerability Endpoints

Safety evaluations in this clinical investigation were meticulously designed to comprehensively assess the incidence, severity, and duration of injection site reactions (ISRs) and adverse events (AEs). ISRs, representing local tissue responses to the injection, were systematically evaluated by the investigator at baseline (Day 0), 2 weeks (Day 14), and 6 weeks (Day 42), employing a standardized grading scale. This scale, ranging from “none” to “light”, “moderate”, and “severe”, provided a quantitative measure of the intensity of specific parameters, including erythema (redness), pain/tenderness, induration (hardening), edema (swelling), papules (bumps), ecchymosis (bruising), pruritus (itching), and discoloration. This standardized approach ensured consistency in reporting and facilitated objective comparisons across subjects and timepoints.

To capture a more comprehensive picture of local tolerability, subjects were provided with detailed daily ISR logs, which they maintained for 6 weeks post-injection. This continuous record allowed for the identification of transient reactions that might have resolved between scheduled investigator visits, providing a more granular understanding of the temporal dynamics of ISRs.

General tolerance, reflecting the overall safety profile of HYDRAGEL A2, was evaluated through the meticulous collection and documentation of all AEs throughout the study period, from the initial screening visit to the final follow-up at Week 6 (Day 42). This comprehensive approach ensured that any systemic or local reactions, regardless of severity or causality, were captured and analyzed. Medical examinations, conducted by the investigator at each visit, included thorough assessments of medical history, verification of inclusion/exclusion criteria, evaluation of local intolerances, documentation of AEs, and recording of concomitant medications. These examinations provided a holistic view of each participant’s health status and potential confounding factors. In cases of premature discontinuation, a final medical examination was performed to ensure subject well-being and to document any AEs potentially related to the study procedures.

The 6-week follow-up period was deemed sufficient to evaluate both the clinical effectiveness and short-term safety of HYDRAGEL A2. This timeframe allowed for the observation and assessment of the majority of expected ISRs and AEs, providing a robust safety profile.

To minimize environmental influences on skin measurements and ensure data consistency, all subjects underwent a standardized acclimatization period of at least 15 min prior to any assessment. This acclimatization was conducted in a controlled environment, maintaining a temperature range of 20–26 °C and a humidity range of 40–60%. This controlled environment minimized variability in skin hydration and temperature, ensuring reliable and reproducible measurements across all subjects.

#### 2.7.3. Injector’s Assessment of Ease of Product Use

The ease of use of the HYDRAGEL A2 product was also evaluated by the injector using a subjective evaluation questionnaire.

### 2.8. Statistical Analyses and Data Presentation

The statistical analysis of this clinical study was conducted with a comprehensive and rigorous approach, employing detailed methodologies to address both efficacy and safety endpoints. A total of 44 female subjects were initially enrolled, but due to two subjects withdrawing their consent, the following data availability was established: Subject CIDP-MRU-0017 had data available up to Day 0 (post-injection), and subject CIDP-MRU-0043 had data available only up to Day-3 (pre-injection). To account for these withdrawals and ensure a robust analysis, three distinct analysis populations were defined:

- Safety Population (n = 43): This population comprised all subjects who received at least one administration of the investigational device, providing a comprehensive assessment of the product’s safety profile.
- Intent-to-Treat (ITT) Population (n = 43): This population included all subjects with at least one post-baseline data point, providing a conservative estimate of intervention effects by including all randomized subjects.
- Per Protocol (PP) Population (n = 42): This population consisted of subjects who completed the study without any major protocol deviations, allowing for an assessment of efficacy under optimal study conditions.

Quantitative variables, representing continuous data, were summarized using descriptive statistics to provide a comprehensive overview of the data distribution. These statistics included the minimum value, maximum value, mean, median, and standard deviation. Qualitative variables, representing categorical data, were summarized using frequencies and percentages to provide insight into the distribution of categorical responses.

For the GAIS, both investigator and subject assessments, reflecting subjective evaluations of aesthetic improvement, were presented as frequencies and percentages across a 5-point scale, ranging from 1 (Very much improved) to 5 (Worse). To facilitate statistical analysis and interpretation, a derived binary parameter was created. This parameter categorized subjects as either “Improved” (i.e., combining “very much improved”, “much improved”, and “improved” responses) or “Not improved” (combining “no change” or “worse” responses). This derived parameter was analyzed using a binomial test for equal proportions (0.5), a statistical test to determine if the proportion of improved subjects differed from 50%, and presented with 95% confidence intervals (CIs) to provide a range of plausible values for the true proportion of improved subjects. A sensitivity analysis, using a global binary variable, was conducted to assess the robustness of the primary efficacy findings. This analysis categorized subjects as “Improved” only if both investigator and subject GAIS scores indicated improvement, providing a more stringent assessment of treatment efficacy.

Instrumental data, obtained from the Antera 3D^®^ (Miravex, Dublin, Ireland), Dermascan^®^ (Cortex, Aalborg, Denmark), Cutometer^®^ (Courage + Khazaka electronic GmbH, Köln, Germany), and Corneometer^®^ (Courage + Khazaka electronic GmbH, Köln, Germany) devices, were analyzed to assess changes in skin texture (roughness), density, elasticity, and hydration, respectively. These data provided objective and quantitative measures of intervention effects. Graphical representations of means with 95% CIs were used to visualize trends and provide a clear depiction of the intervention’s impact on skin parameters. The percentage change from baseline (Day -3) was calculated for each parameter to quantify the magnitude of change over time. The evolution across time, reflecting changes in skin parameters from baseline to follow-up visits, was analyzed using paired t-tests or Wilcoxon signed-rank tests. The choice between these tests depended on the normality of data distribution, as assessed by the Shapiro–Wilk test at a 1% significance level. Paired t-tests were used for normally distributed data, while Wilcoxon signed-rank tests were used for non-normally distributed data.

A significance level of *p* ≤ 0.05 was used for all statistical tests, indicating that results with a *p*-value less than or equal to 0.05 were considered statistically significant. Statistical tests were conducted using SPSS 19.0 and Microsoft Excel (Microsoft Corporation, Redmond, WA, USA) for general statistical analyses, and GraphPad Prism v.8.0.2 (GraphPad Software, San Diego, CA, USA) for graphical representations. Detailed levels of statistical significance are presented in the Results section and in the Supplementary Materials tables. Abbreviations such as S (Significant), LS (Limit Significant), NS (Not Significant), and CI (Confidence Interval) were used to denote statistical outcomes, providing a concise summary of the statistical findings.

## 3. Results and Discussion

### 3.1. Global Aesthetic Improvement Scales

**Figure 1:**
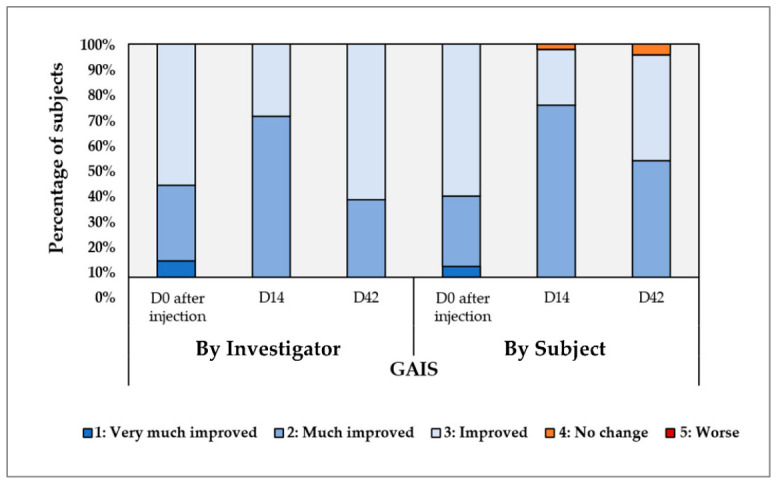
Results of both GAIS scores. Details of the definitions of the various grades are presented in Table S1. Statistical analysis results are presented in Table S2. IGAIS, investigator global aesthetic improvement scale; SGAIS, subject global aesthetic improvement scale.

The investigator and subjects graded the improvement level for the overall face appearance using the Global Aesthetic Improvement Scale (GAIS) on D0 after injection, D14, and D42. The results are presented in Figure 1.

Immediately following the injection (Figure S1), the investigator observed a 100% response rate, classifying all subjects as exhibiting clinical improvement. Of note, the mean volume injected per cheek was 1.4 mL. This initial improvement was maintained throughout the study period, persisting until the final evaluation at Day 42. This consistently high proportion of improved subjects, starting with 100% immediately post-injection (Day 0) and exhibiting a minor decline at subsequent time points (i.e., 97.6% at Days 14 and 95.2% at Days 42), suggests a robust and sustained post-injection effect of the investigated device. For the aesthetic practitioner, this minor statistical decline from Day 0 to Day 42 accurately captures the well-known ‘biostimulator gap.’ Immediately post-procedure, patients benefit from the combined effects of the HA’s osmotic properties and transient procedure-related edema, which may contribute to the high rate of perceived global improvement. As this initial fluid retention normalizes over the following weeks, some patients may report a transient perception of reduced volume before longer-term tissue changes become clinically appreciable. The high level of improvement maintained at Day 42 suggests sustained aesthetic benefit, which may reflect a combination of hydration effects and progressive tissue remodeling. Furthermore, subjective assessments, gathered via a patient-reported questionnaire at Days 14 and 42, corroborated the investigator’s findings, revealing perceived enhancements in various skin parameters (Table 3).

The congruence between objective investigator assessments and subjective patient-reported outcomes strengthens the assertion of the treatment’s clinical efficacy. The 100% initial subjective satisfaction indicates that the uncrosslinked, high-molecular-weight biopolymer complex provides immediate structural enhancement without triggering adverse tactile feedback or sensory foreign-body sensations, ensuring the tissue feels as natural as it looks. Notably, the proportion of subjects reporting “Agreement” or “Totally Agreement” with perceived improvements in skin condition demonstrated a relevant increase between Day 14 and Day 42 (Table 3). In the context of facial rejuvenation, the subjective reporting of a ‘Decrease of skin fine lines’ by 85.7% of subjects (combining ‘Agree’ and ‘Totally agree’ responses) at Day 42 highlights a critical therapeutic niche. The ability of this injectable biopolymer complex to smooth these fine lines purely through bio-structural redensification provides practitioners with an invaluable tool for patients seeking highly natural, dynamic aesthetic outcomes.

The described temporal trend suggests a progressive enhancement of perceived skin quality over the observation period. It is clinically vital to interpret the self-reported ‘Lifting effect’—noted by over 80% of subjects at both Day 14 and Day 42—through the lens of superficial skin redensification. Because HYDRAGEL A2 utilizes non-crosslinked HA and is injected into the dermis or superficial subcutaneous tissue, it does not provide true structural, volumizing lift. Instead, this patient-perceived ‘lift’ is the subjective manifestation of enhanced tissue turgor and dermal tightening. Patients routinely perceive this reduction in superficial laxity and increased ‘snap-back’ as a lifting effect, which is a powerful driver of the high satisfaction rates observed.

Furthermore, the percentage of subjects expressing satisfaction with the injection results also exhibited an increase, indicating a growing sense of overall positive intervention outcome over time.

The consistent upward trend in both perceived improvement and overall satisfaction underscores the sustained and cumulative benefits of the intervention. A critical metric for practicing injectors within the subjective questionnaire data is the complete absence of negative responses. In the highly subjective field of aesthetic medicine, where patient expectations are notoriously difficult to manage, achieving a ‘zero non-responder’ floor for perceived worsening or dissatisfaction is notable. This provides clinicians with high confidence that integrating HYDRAGEL A2 into their practice carries a very low risk of patient buyer’s remorse, securing high patient retention and procedural satisfaction.

Beyond the objective physical improvements, the progressive increase in subjective satisfaction highlights the psychological value of the intervention. In aesthetic medicine, instrumental success must align with subjective patient perception to be considered clinically meaningful. The temporal alignment of self-reported reductions in fine lines, improved pore aspect, and enhanced lifting effect—culminating in 95.2% of subjects reporting satisfaction at Day 42—suggests that the combined effects of PN, HA, and niacinamide effectively translate into a visible rejuvenation that drives high patient confidence and adherence. This emphasizes the formulation’s utility not just as a structural filler, but as a comprehensive skin quality enhancer.

### 3.2. Antera 3D Results for Skin Texture and Roughness

**Figure 2:**
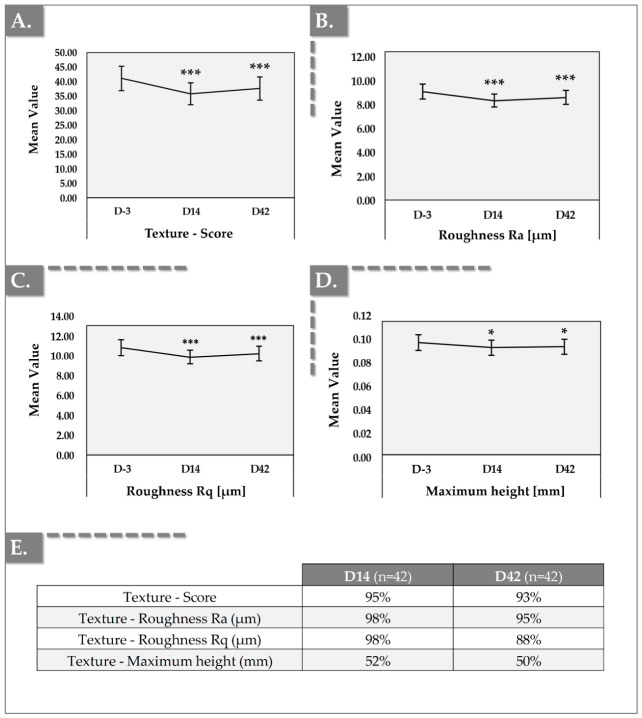
Clinical monitoring results using the Antera 3D device. (**A**) Texture score evolution. (**B**) Roughness “Ra” parameter evolution. (**C**) Roughness “Rq” parameter evolution. (**D**) Maximum height evolution. (**E**) Proportion of the subjects showing improvement across the different parameters. Error bars = 95% confidence interval. Statistical significance (i.e., *** or *p*-value ≤ 0.001; * or *p*-value ≤ 0.05) was evidenced by asterisks. Detailed statistical analysis results are reported in Table S3.

Image acquisition of skin texture on both cheeks was performed on D-3, D14, and D42 and were analyzed (Figure 2). The findings of this study provide compelling evidence that the investigated device induced a significant amelioration of skin texture. This is substantiated by statistically significant reductions in texture scores and roughness parameters (Ra and Rq) observed at both Day 14 and Day 42. For the aesthetic clinician, the specific reduction in the Rq parameter (root mean square roughness) holds unique clinical value. While Ra averages out overall surface texture, Rq is mathematically much more sensitive to extreme peaks and deep valleys in the skin’s micro-topography. Notably, the most substantial improvements were recorded at Day 14, followed by a modest attenuation of these effects by Day 42.

However, it is crucial to emphasize that, even at Day 42, the measured improvements remained statistically significant when compared to baseline values, indicating a sustained, albeit slightly diminished, benefit (Figure 2). In the clinical setting, this statistically significant reduction in both arithmetic (Ra) and root mean square (Rq) roughness translates directly into the aesthetic concept of ‘skin radiance’ or ‘glow.’ While deep structural fillers correct volumetric shadowing, superficial skin quality relies heavily on the uniformity of light reflection across the stratum corneum. By smoothing these micro-irregularities and reducing superficial crepiness, the combined HA and PN hydrogel alters the skin’s optical properties. The smoother surface allows for specular (mirror-like) light reflection rather than diffuse light scattering, which clinically manifests as the highly desirable, radiant complexion that likely drove the 100% immediate patient satisfaction reported on the GAIS at Day 0.

**Figure 3:**
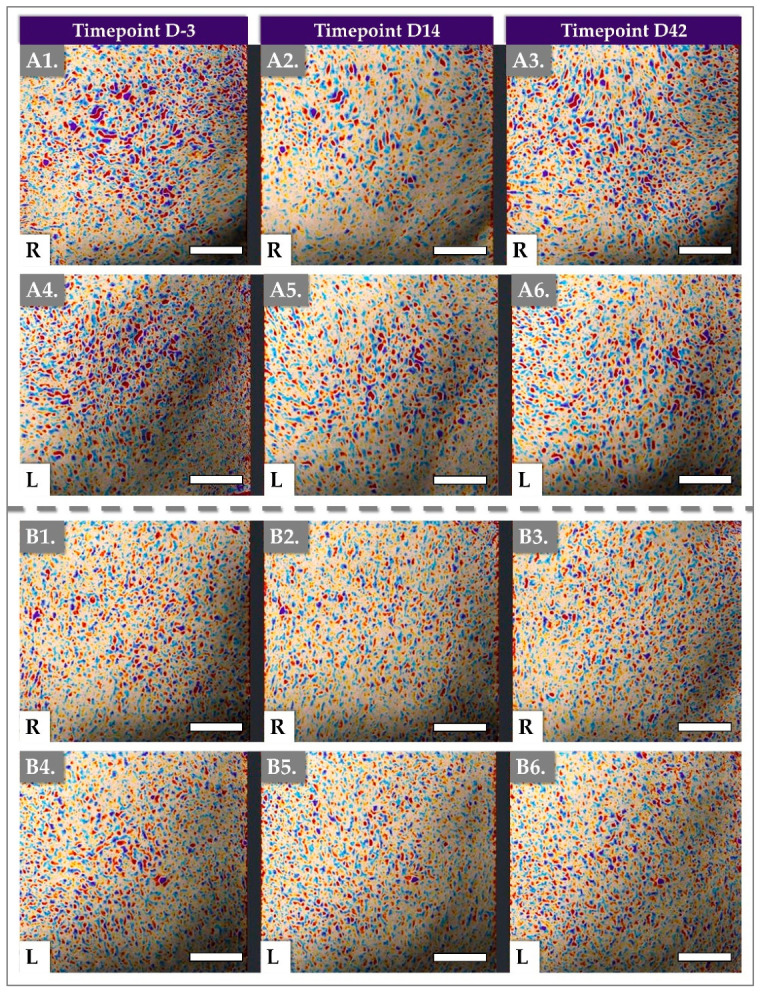
Results of Antera 3D imaging at various timepoints of the clinical study. (**A1**–**A3**) Imaging of the right cheek (R) for patient N°7. (**A4**–**A6**) Imaging of the left cheek (L) for patient N°7. (**B1**–**B3**) Imaging of the right cheek for patient N°18. (**B4**–**B6**) Imaging of the left cheek for patient N°18. Scale bars = 5 mm.

Representative Antera 3D imaging is presented in Figure 3.

### 3.3. DermaScan Results for Dermal Structure and Thickness

**Figure 4:**
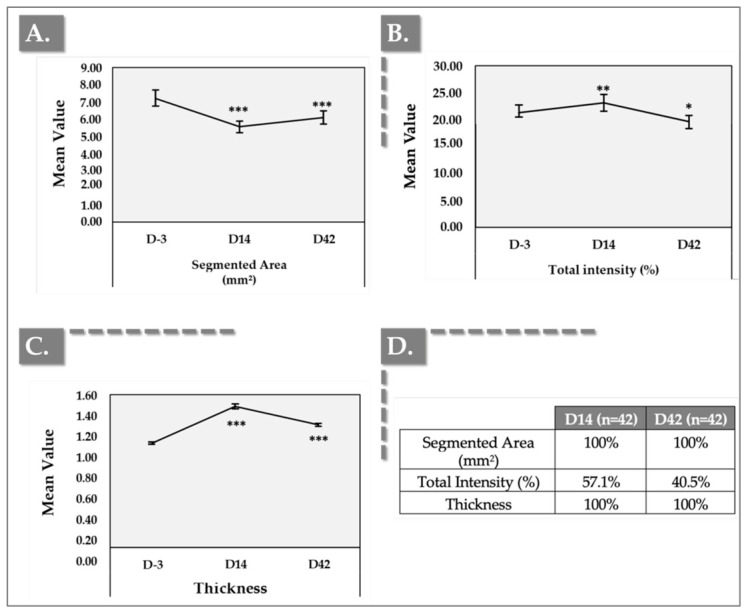
Clinical monitoring results using the Dermascan device. (**A**) Segmented area value evolution. (**B**) Total intensity parameter evolution. (**C**) Thickness parameter evolution. (**D**) Proportion of the subjects showing improvement across the different parameters. Error bars = 95% confidence interval. Statistical significance (i.e., *** or *p*-value ≤ 0.001; ** or *p*-value ≤ 0.010; * or *p*-value ≤ 0.05) was evidenced by asterisks. Detailed statistical analysis results are reported in Table S4.

**Figure 5:**
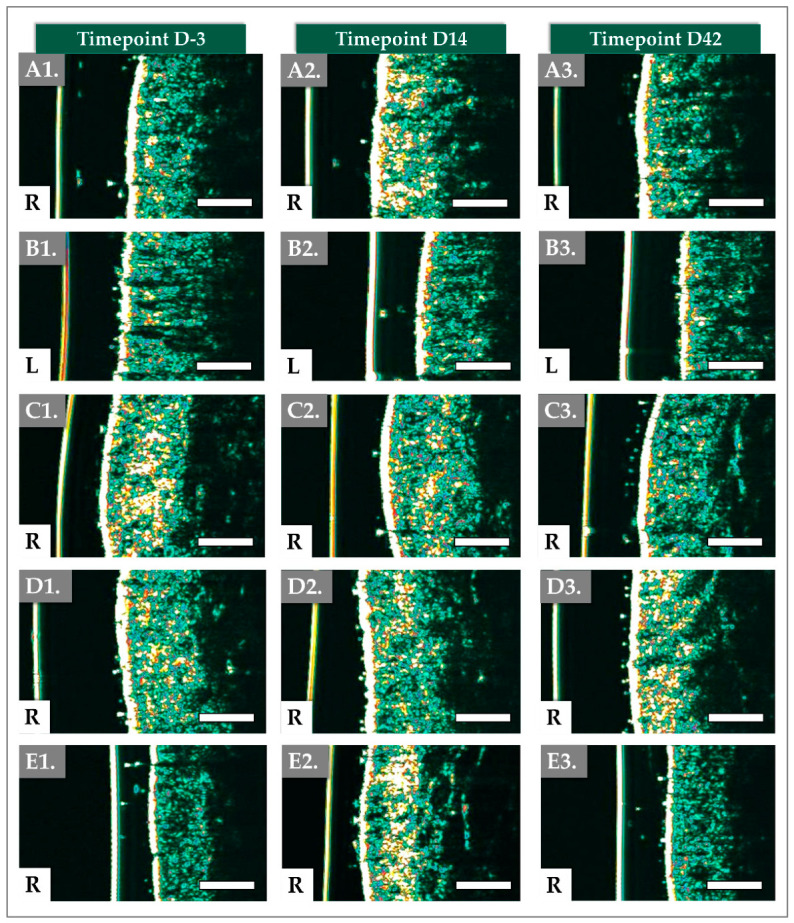
Results of Dermascan imaging at various timepoints of the clinical study. (**A1**–**A3**) Imaging of the right cheek (R) for patient N°18. (**B1**–**B3**) Imaging of the left cheek (L) for patient N°25. (**C1**–**C3**) Imaging of the right cheek for patient N°40. (**D1**–**D3**) Imaging of the right cheek for patient N°9. (**E1**–**E3**) Imaging of the right cheek for patient N°19. Scale bars = 2 mm.

Ultrasound images were taken using the Dermascan on both cheeks at D-3, D14, and D42 and the images were analyzed (Figure 4 and Figure 5).

In ultrasound imaging, the “segmented area” denotes a precisely delineated region of interest within the dermis, isolated for targeted analysis. Longitudinal monitoring of these specific areas enables the quantification of changes in collagen density within the targeted region, thereby providing objective evidence of enhanced skin firmness and resilience.

Total Intensity, a quantitative measure of the echogenic response from these segmented areas, serves as a clinical proxy for collagen and structural protein density. Consequently, it offers a valuable metric for assessing the treatment’s efficacy in augmenting skin thickness, firmness, and resilience. Our results demonstrate that the product significantly reduced the segmented area of concern while concurrently increasing skin thickness, exhibiting both immediate and sustained effects.

For the aesthetic clinician, this objective, ultrasound-verified increase in skin thickness serves as the structural foundation for the patient-reported cosmetic outcomes. In the subjective evaluations, the proportion of subjects reporting agreement regarding the ‘Decrease of skin fine lines’ reached 69% by Day 14 and was maintained at 85.7% by Day 42 (Table 3). Clinically, this is one of the macroscopic manifestations of the Dermascan findings.

The initial elevation in Total Intensity observed at Day 14 suggests an early enhancement of skin structural properties. From an ultrasonic physics perspective, this Day 14 spike in Total Intensity is largely driven by the specific acoustic impedance of the highly hydrated 1.8 MDa HA network combined with the dense 1.5 MDa PN chains. Ultrasound waves reflect strongly at the interfaces of tissues with differing densities. However, a subsequent, albeit modest, decrease was noted at Day 42. This specific macroscopic thickness trajectory is a classic hallmark of the hybrid biomaterial’s integration kinetics. The pronounced peak in dermal thickness at Day 14 is predominantly hydro-mechanical; it reflects the maximal water-binding saturation of the 1.8 MDa HA polymer chains before enzymatic degradation occurs. As this fluid-phase volumization naturally recedes by Day 42, the thickness metric slightly decreases. However, the fact that thickness remains significantly elevated above baseline at Week 6 indicates a successful phase transition.

Nonetheless, the overall findings underscore the device’s capacity to induce favorable modifications in key skin parameters and maintain clinically relevant benefits throughout the observation period.

### 3.4. Cutometer Results for Skin Elasticity and Firmness

**Figure 6:**
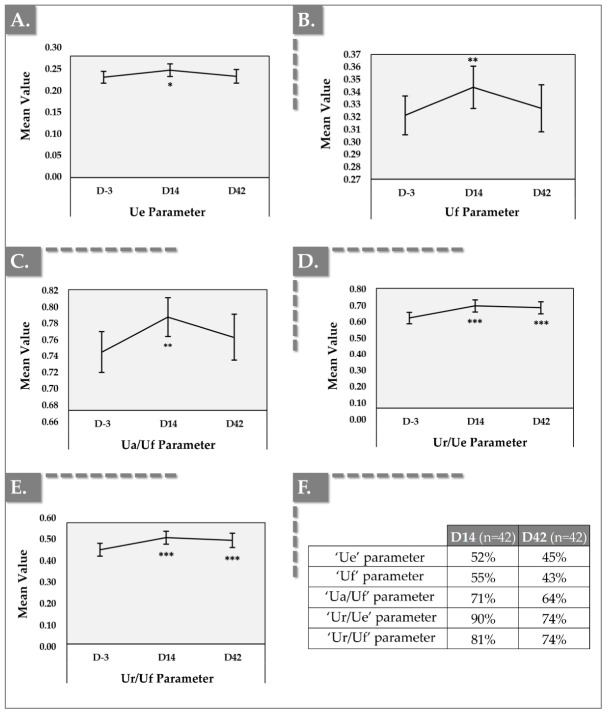
Clinical monitoring results using the Cutometer device. (**A**) “Ue” parameter evolution. (**B**) “Uf” parameter evolution. (**C**) “Ua/Uf” parameter evolution. (**D**) “Ur/Ue” parameter evolution. (**E**) “Ur/Uf” parameter evolution. (**F**) Proportion of the subjects showing improvement across the different parameters. Error bars = 95% confidence interval. Statistical significance (i.e., *** or *p*-value ≤ 0.001; ** or *p*-value ≤ 0.010; * or *p*-value ≤ 0.05) was evidenced by asterisks. Detailed statistical analysis results are reported in Table S5.

The measurement of skin firmness/elasticity was performed on both cheeks on D-3, D14, and D42 using a Cutometer^®^ dual MPA 580 (Courage + Khazaka electronic GmbH, Köln, Germany) (Figure 6).

The parameter Ue, defined as immediate deformation or skin extensibility, quantifies the skin’s initial response to applied force. It reflects the skin’s capacity to stretch or yield upon initial stress. A lower Ue value typically indicates firmer skin that exhibits greater resistance to initial deformation, while a higher Ue value suggests increased skin flexibility, potentially indicative of reduced firmness.

The parameter Uf, representing final distension or skin distensibility, provides insight into the skin’s passive response to sustained force and has implications for skin firmness. It reflects the skin’s ability to deform under prolonged stress. Lower Uf values suggest tighter skin, potentially signifying enhanced structural integrity.

The ratio Ua/Uf, termed gross elasticity, encompasses both elastic and viscous deformation components. It represents the skin’s overall elasticity, including creep and creep recovery. Elevated Ua/Uf values indicate heightened skin elasticity, reflecting a greater ability to return to its original form after stretching. An increase in this Ua/Uf ratio is generally considered favorable, signifying improved elasticity.

The ratio Ur/Ue, representing net elasticity, isolates the elastic component of skin deformation, excluding viscous effects. It quantifies the skin’s immediate recovery capacity. Higher Ur/Ue values suggest a robust capacity for immediate recovery, which is indicative of resilient, elastic skin. An increase in the Ur/Ue ratio is favorable, signifying enhanced skin elasticity and firmness.

The ratio Ur/Uf, termed biological elasticity, assesses the skin’s recovery relative to the total deformation. It reflects the skin’s ability to return to its original shape after significant stretching. Elevated Ur/Uf values indicate a strong recovery capacity, reflecting improved elasticity. An increase in this ratio is particularly favorable in cosmetic trials targeting skin elasticity and firmness [7]. Fundamentally, these Cutometer^®^ metrics demonstrate a beneficial shift in the skin’s viscoelastic balance. Aging skin typically becomes more viscous (i.e., fluid-like, prone to permanent deformation and sagging) and less elastic (i.e., solid-like, capable of immediate rebound). The concurrent improvements in net elasticity (Ur/Ue) and biological elasticity (Ur/Uf) indicate that the HYDRAGEL A2 intervention successfully reverses this trend.

Overall, the results of this study demonstrate that the product induced statistically significant improvements in skin elasticity and firmness by Day 14, with sustained benefits observed for Ur/Ue and Ur/Uf through Day 42 when compared to baseline (Day-3). Notably, both absolute (Ue, Uf) and relative (Ur/Ue, Ur/Uf, Ua/Uf) parameters exhibited positive trends, collectively reflecting the device’s efficacy in enhancing facial skin mechanical properties.

### 3.5. Corneometer Results for Skin Hydration

**Figure 7:**
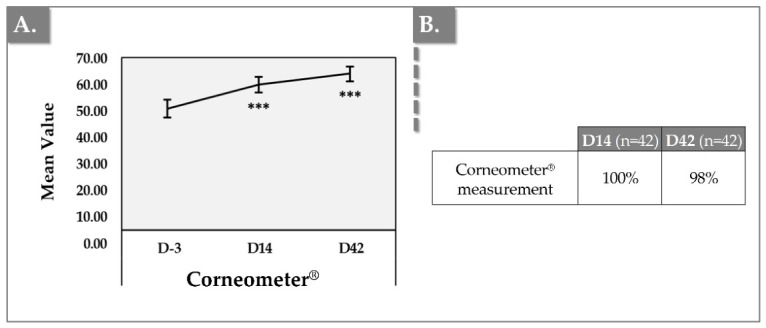
Clinical monitoring results using the Corneometer device. (**A**) Skin hydration level evolution. (**B**) Proportion of the subjects showing improvement across the different parameters. Error bars = 95% confidence interval. Statistical significance (i.e., *** or *p*-value ≤ 0.001) was evidenced by asterisks. Detailed statistical analysis results are reported in Table S6.

The hydration level of the skin was measured on both cheeks using the Corneometer^®^ CM 825 on D-3, D14, and D42 (Figure 7).

There is a statistically significant increase in hydration levels at both D14 and D42 (59.15 ± 10.22 and 63.52 ± 9.64, respectively) when compared to D-3 (49.39 ± 11.65) (*p* < 0.001), which shows a notable improvement over time (i.e., 28.60% increase between D-3 and D42) (Figure 7). Crucially, this objective biophysical data is perfectly mirrored by the patients’ own tactile experiences, successfully bridging the gap between instrumental science and clinical reality. According to the subjective questionnaires, exactly 100% of the cohort reported an improvement in skin hydration by Day 42, combining ‘Agree’ (59.5%) and ‘Totally agree’ (40.5%) responses (Table 3). In aesthetic dermatology, objective instrumental gains are only clinically relevant if the patient can physically perceive them. The absolute concordance between the statistically significant Corneometer readings and the unanimous subjective patient validation confirms that the deep dermal integration of HA, PNs, and niacinamide successfully translates into a tangible, macroscopic improvement in the skin’s surface moisture barrier.

The persistence of this statistically significant hydration peak at Day 42 strongly indicates that the observed effect may have shifted from exogenous to endogenous. Given the rapid in vivo turnover of non-crosslinked HA, the injected hydrogel’s direct osmotic pull is substantially diminished by week six. Furthermore, the consistent standard deviations observed in these Corneometer measurements across the cohort signify a highly homogenous biodistribution of the hydrogel within the tissue. If the HA and PN matrix were prone to clumping or uneven integration within the interstitial fluid, it would result in localized patches of high hydration and vast standard deviations across the measurement zones. This tight data spread provides biophysical confirmation that the formulation’s rheology permits smooth, uniform diffusion throughout the targeted dermal plane, minimizing the risk of localized fluid pooling.

### 3.6. Safety and Injectability Evaluation Results

This study meticulously documented the incidence and severity of injection site reactions (ISRs) at multiple timepoints (Day 0, Day 14, and Day 42), with separate evaluations conducted for the right and left sides of the face. The presence of mild, moderate, or severe reactions was collectively categorized as “Presence.” Immediately following the injection (Day 0), the most frequently observed ISRs, as reported by both the investigator and subjects, included erythema, pain/sensitivity, induration/firmness, edema, and papules, which were all common and expected. It is essential for practitioners to guide patients through the perceptual transition from acute procedural ‘induration’ to true biological ‘firmness’. While patients often misinterpret the Day 0 injection-related edema and fluid-bound induration as the final volumizing result, this acute phase spontaneously resolves by Day 14. Remarkably, exactly as this transient procedural swelling clears, the subjective questionnaire reveals that 81% of subjects reported a true improvement in skin firmness at Day 14. Proactively educating patients that the initial firmness is a temporary reaction to the hydrogel’s osmotic pull, whereas the firmness felt at Day 14 and beyond represents genuine structural redensification, is vital for managing aesthetic expectations and preventing post-treatment anxiety. Notably, these reactions had spontaneously resolved completely by the follow-up visits at Day 14 and Day 42 (Table S7). In addition to the investigator’s observations, the subjects independently recorded their experiences in a personal ISR log, reporting a broader spectrum of signs, including erythema, pain/sensitivity, induration/firmness, edema, papules, ecchymosis, pruritus, and discoloration (Tables S7 and S8). Importantly, no serious adverse events (SAEs) of any kind were documented throughout the study period. For the practicing clinician, the complete absence of SAEs over the 42-day observation period is perhaps the most vital metric for practice integration.

As regards investigational device ease of use, the injector answered a subjective evaluation questionnaire on the overall quality of the injection process on D0. The results are presented in Table 4.

For all subjects, the investigator was very satisfied/satisfied with the ease of hydrogel injection, and the procurement of immediate aesthetic results on D0. This high injector satisfaction—with 86.0% reporting ‘very satisfied’ for injection—carries clinical weight beyond mere procedural convenience. When injecting superficially into the dermis to improve skin quality, precision is paramount. A hydrogel that requires high, variable, or stuttering extrusion push the practitioner to compromise their micro-droplet or linear threading technique, inevitably leading to localized product pooling, visible blebs, or unequal biostimulatory distribution. The smooth, predictable tactile feedback reported here confirms that the hydrogel’s rheology supports the fluid, continuous cannula movement required for flawless, homogenous superficial tissue redensification.

### 3.7. General Discussion

#### 3.7.1. Combining Injectable Biopolymers for Facial Skin Redensification and General Rejuvenation

The visible manifestations of facial aging, a confluence of intrinsic chronological processes and extrinsic environmental insults, present a significant challenge in aesthetic medicine. This complex phenomenon, characterized by diminished skin elasticity, dehydration, textural irregularities, and altered pigmentation, necessitates a multifaceted approach [8,9,30]. The escalating demand for minimally invasive aesthetic interventions has driven innovation in injectable device technologies, aiming to restore youthful skin quality [13,18,21]. This study evaluated a novel combination of hyaluronic acid (HA), polynucleotides (PN), and niacinamide delivered via injection, assessing its capacity to induce facial skin redensification and overall rejuvenation. This strategy addresses a critical need in clinical practice: the ability to simultaneously target diverse aging pathways with a single, streamlined formulation, potentially improving patient compliance and reducing procedural burden.

HA, a naturally occurring polysaccharide, forms the foundational element of the investigational formulation. Its exceptional water-binding capacity directly addresses skin dehydration, a hallmark of aging [31,32]. Furthermore, HA interacts with cellular receptors, stimulating fibroblasts, thereby promoting dermal matrix (e.g., collagen, elastin) remodeling [31,32]. This dual action, namely hydrating and stimulating, contributes to improved skin volume and elasticity, counteracting age-related structural decline.

Niacinamide, a hydrosoluble vitamin B3 derivative, is a well-established excipient in injectable hydrogel formulations [23]. Within the HYDRAGEL A2 matrix, niacinamide serves a dual physicochemical function: it contributes to the preservation of the HA polymer network by scavenging ROS that would otherwise accelerate hydrogel depolymerization, and it supports the maintenance of optimal hydration levels within the scaffold structure [23,33,34,35,36]. Niacinamide has been widely incorporated into commercially available injectable hydrogel devices, including Louna Fillers, Innovyal Regenerative Action, and NCTF 135HA, where it functions as a matrix-stabilizing component [35,36].

PNs, high-molecular-weight biopolymers obtained through controlled purification of DNA fragments from fish-derived raw material, are incorporated into the formulation to enhance its rheological and structural properties [35]. As macromolecular chains, PNs increase the viscosity of the hydrogel complex, contributing to improved tissue scaffolding capacity and support within the dermal compartment [35]. The combination of HA, PNs, and niacinamide within a single hydrogel matrix is designed to optimize the overall physical performance of the device, including enhanced structural integrity, increased resistance to in situ degradation, and sustained scaffolding capacity. This integrated system provides a durable physical substrate that supports the skin’s natural homeostatic processes while contributing to a more homogeneous and stable tissue architecture. In this context, their ability to promote dermal matrix remodeling at the structural level may further improve tissue organization, potentially amplifying the intended rejuvenating effect, as shown in several studies [35,37,38,39].

The direct conjoint delivery of these functional ingredients into the dermal layer, via injection, offers a significant advantage over topical applications. Specifically, this method ensures optimal bioavailability, bypassing the skin’s natural barrier and delivering molecules directly to their respective target sites. This approach is particularly relevant for HA and PNs, whose penetration through the skin’s outer layer can be limited due to their high molecular weight [22,39,40]. Additionally, the incorporation of niacinamide into an HA-based gel may also extend the longevity of the HA network itself, as its antioxidant properties can significantly protect HA from degradation [36].

Overall, the combined application of PNs with HA and niacinamide represents a holistic approach to facial rejuvenation, aiming at addressing both superficial and deep structural changes. This strategy targets the key mechanisms responsible for skin health, potentially yielding more comprehensive and longer-lasting aesthetic outcomes. The established safety profiles of injectable niacinamide, coupled with the biocompatibility of PNs, support the clinical viability of this approach.

#### 3.7.2. The Biophysical Properties of HYDRAGEL A2

Injectables incorporating HA, PNs, and niacinamide exhibit a synergistic interplay of biophysical, chemical, and biochemical properties that contribute to enhanced dermal rejuvenation. HA, a glycosaminoglycan, provides immediate volumization and hydration through its exceptional water-binding capacity, restoring skin turgor and diminishing fine lines. PNs, composed of long chains of nucleotides, contribute to the gel’s properties beyond simple volumization [35]. PNs significantly influence the rheological properties of a given dermbooster. HYDRAGEL A2 is composed of high MW hyaluronic acid (i.e., MW 1.8 MDa) at a concentration of 7.5 mg/mL and high MW polynucleotides (i.e., MW 1.5 MDa) at a concentration of 11.25 mg/mL. The selection of these specific high-MW profiles is biologically highly deliberate. While low-molecular-weight HA fragments are known to occasionally trigger pro-inflammatory pathways and undergo rapid degradation, high MW HA (1.8 MDa) is recognized by the body as native, exerting anti-inflammatory and tissue-soothing effects [32]. Similarly, the 1.5 MDa high-molecular-weight PNs provide an enhanced matrix support function from within [35]. The biopolymers increase the gel’s viscosity and viscoelasticity, resulting in a more robust scaffolding effect within the dermal matrix (Figure S2). Of note, the storage modulus (G′) of HYDRAGEL A2 has a mean value of 76.1 Pa and the loss modulus (G″) has a mean value of 50.3 Pa. This enhanced structural support improves the product’s ability to resist deformation and maintain its shape, leading to more sustained skin structural support and lifting properties in vivo.

For the aesthetic investigator, these specific viscoelastic parameters translate directly into predictable tissue integration. Unlike heavily crosslinked volumizers designed to resist deformation and remain highly localized, this moderately elastic yet spreadable profile facilitates smooth, homogeneous distribution within the targeted dermal layers without the risk of visible surface irregularities or the Tyndall effect. This rheological balance ensures that the regenerative active ingredients are dispersed evenly across the aesthetic zones, maximizing the biostimulatory surface area. Clinically, these rheological changes directly impact product attributes, resulting in improved structural integrity, which can in turn lead to more controlled and precise placement within the targeted tissue layers (i.e., dermis or superficial fat compartments), optimizing aesthetic outcomes [41,42].

Biochemically, several studies have reported that polynucleotides (PNs) can stimulate fibroblast proliferation and promote collagen and elastin synthesis [20,22,27,35]. In particular, a formulation closely matching the PN/HA/B3 composition has been shown in vitro to increase collagen production and fibroblast activity. This process may improve the dermal matrix’s structural integrity and elasticity. Furthermore, PNs and niacinamide exhibit antioxidant and anti-inflammatory properties, mitigating oxidative stress and reducing inflammation, both of which are critical factors in skin aging and general tissue health [20,22,23,24,43,44]. Evidence supporting the anti-inflammatory and antioxidative effects of PNs has also been reported in other medical fields, including osteoarthritis management [45,46].

Generally, the combined effect of these components creates a multifaceted rejuvenation strategy. HA provides immediate hydration and rheological structure, PNs may stimulate long-term structural improvement while modulating rheology, and niacinamide optimizes the dermal environment, prolonging the longevity of the HA gel by mitigating degradation and fostering a healthier cellular milieu. The specific rheological properties of such combined fillers are crucial determinants of their tissue integration, post-administration biodistribution, and the resulting aesthetic outcomes.

#### 3.7.3. Linking Product Formulation Attributes with Clinical Results

The compelling clinical outcomes observed in this study, demonstrating statistically significant and sustained improvements in skin quality following HYDRAGEL A2 injections, are a direct testament to the synergistic interplay between its constituent ingredients and the engineered biophysical attributes of the hydrogel. The immediate aesthetic enhancements, as evidenced by the 100% improvement rate on the GAIS at Day 0, are primarily attributable to the rapid mechanical and hydrating effects of HA. This immediate plumping effect is not merely cosmetic; it also serves to enhance patient satisfaction by providing tangible, visible results shortly after the procedure. Even at Day 42, 95.2% of patients reported an improvement (Figure 1). However, it is vital to distinguish this acute mechanical volumization from physiological tissue redensification. The 100% improvement recorded at Day 0 primarily reflects the physical presence and water-binding capacity of the injected hydrogel rather than structural dermal rejuvenation. True biological remodeling requires a more extended period to manifest clinically.

Although the GAIS is not a fully objective measurement method, it remains the gold standard in aesthetic medicine for assessing global aesthetic outcomes. Notably, these results appear relatively high compared with other injectable non-crosslinked gel formulations designed to improve skin quality [18,47]. Specifically, in two recent studies evaluating skin quality improvement, one using a formulation composed of high molecular weight, non-crosslinked HA supplemented with amino acids (i.e., arginine, glycine, lysine, proline, and valine), biotin, riboflavin, and vitamins C and E (CELLBOOSTER^®^ Lift, Suisselle, Zug, Switzerland) and another using a formulation of non-crosslinked HA combined with sorbitol (2.6% H-HA/3.2% sorbitol), the reported outcomes were lower in comparison [18,47].

The sustained improvements in skin texture, as objectively quantified by the significant reductions in roughness parameters (Ra and Rq) measured using Antera 3D^®^ imaging, are particularly noteworthy. Ra, representing the arithmetic average roughness, and Rq, the root mean square roughness, both exhibited statistically significant reductions at Days 14 and 42, indicating a progressive skin smoothing effect. Furthermore, the statistically significant reduction in the “maximum height” parameter provides important evidence of improved skin surface homogeneity following injection. In cases where high-viscosity biopolymers fail to distribute uniformly within the tissue, localized elevations or irregularities may occur, which can be reflected by increased topographical peaks. The observed decrease in this parameter suggests that the HYDRAGEL A2 formulation, likely due to its shear-thinning behavior, promotes a more even distribution within the treated area, resulting in a smoother and more homogeneous skin surface with reduced prominence of pores and textural irregularities. The sustained improvement suggests a complex interplay of regenerative processes initiated by the combined actions of HA, PNs, and niacinamide. Additionally, this sustained statistical significance at Day 42 serves as in vivo clinical evidence of niacinamide’s macromolecular shielding effect. Typically, non-crosslinked, linear HA used as a monotherapy is highly susceptible to rapid depolymerization by ROS and hyaluronidases, often leading to a complete regression of textural benefits well before the 6-week mark [17]. The robust retention of the smoothed micro-relief (Ra and Rq parameters) at Day 42 strongly suggests that the 15 mg/mL concentration of niacinamide successfully mitigated localized oxidative stress, thereby extending the half-life and mechanical scaffolding capability of the 1.8 MDa HA network long enough for the PN to properly integrate into the dermis. From a clinical perspective, these objective instrumental reductions in micro-topographical roughness (Ra and Rq) correlate directly with macroscopic aesthetic improvements, particularly the effacement of superficial rhytides [48]. This Antera 3D^®^ data robustly validates the patient-reported outcomes, where a substantial proportion of subjects noted a visible decrease in their fine lines by Day 14 and Day 42. By rebuilding the dermal ECM, HYDRAGEL A2 provides the structural ‘fill’ necessary to smooth these surface irregularities, proving that the formulation effectively targets the structural deficits underlying visible wrinkle formation. Lee et al. (2020) reported, in a split-face study, comparable effects between an HA-based formulation and a high MW PN-based formulation (i.e., Rejuran^®^; PharmaResearch Products Co., Ltd., Gangneung-si, South Korea) in terms of skin hydration and elasticity [49]. However, a significant difference was observed for skin roughness, favoring the PN formulation [49]. While the improvement in skin roughness gradually decreased over time in the HA-treated areas, it tended to increase and remain sustained in the PN-treated areas [49]. This effect may be largely attributed to the activity of PN, whose gradual enzymatic degradation may create favorable conditions in the dermis that support tissue regeneration. Additionally, Lampridou et al. (2025), in a systematic review assessing the effectiveness of polynucleotides in aesthetic medicine, identified four studies reporting improvements in skin texture following injections of PN-based formulations [50].

Dermascan^®^ imaging, which provided objective assessments of skin thickness and density, revealed significant structural improvements attributable to the combined action of HA and PNs. Dermal density (i.e., total intensity and segmented area values in Figure 4), which reflects the structural organization of the ECM, including collagen, elastin, and HA, represents an important parameter for evaluating tissue remodeling following regenerative procedures [51]. Of note, total intensity refers to the overall echogenic signal within the selected region of interest (ROI) of the ultrasound image. Higher total intensity generally indicates greater dermal density and more organized collagen structures. The segmented area corresponds to the portion of the dermis identified by the software as echogenic (dense) tissue within the selected ROI [52]. In the present study, dermal density showed a significant increase at all follow-up visits compared with the baseline, supporting the hypothesis that injection of the HYDRAGEL A2 device induces measurable structural changes within the dermal compartment after only one injection session. Additionally, the mean volume injected per cheek was relatively low at 1.4 mL.

Dermal density is commonly assessed using high-frequency ultrasound imaging techniques, such as performed with the Dermascan^®^, which allow non-invasive visualization and quantitative evaluation of dermal echogenicity. Interestingly, clinical studies investigating improvements in dermal density with non-crosslinked HA formulations typically require multiple treatment sessions to achieve significant outcomes. For example, protocols involving NCTF^®^135HA (Filorga Laboratories, Paris, France) generally include three injection sessions, Tri-Hyal technology has been evaluated using two injection sessions, Rejuran^®^ (PharmaResearch Products Co., Ltd., Gangneung-si, South Korea) and Yvoire-Hydro (LG Chem, Seoul, South Korea) has been evaluated in a split face study using three injection sessions, and Cellbooster Lift protocols commonly involve three injection sessions. These observations suggest that repeated administration may be necessary to induce measurable ECM remodeling when using non-crosslinked HA-based formulations [18,49,51,53]. Such findings are consistent with the concept that HA-based biomaterials may promote progressive dermal remodeling through improvements in ECM organization [18,21,51,53]. The increased total intensity in segmented area and thickness suggests an increase in dermal density, which can be attributed to increased collagen deposition and improved ECM organization.

While instrumental data such as Dermascan^®^ and Cutometer^®^ readings offer robust quantitative validation of this ECM organization, standardizing the visual representation of these improvements is crucial for clinical communication. The visual improvements captured via the standardized VISIA-CR^®^ 2D photography (Canfield Scientific, Parsippany, NJ, USA) (Figure S3) directly corroborate the microscopic dermal changes. Demonstrating exactly how an instrumental reduction in roughness or an increase in dermal thickness manifests in a standardized photograph as a visibly smoother, more uniform skin contour provides the relatable evidence that aesthetic professionals utilize in daily practice.

Cutometer^®^ measurements, which objectively assessed skin elasticity and firmness, revealed statistically significant improvements in Ur/Ue and Ur/Uf parameters, with sustained benefits observed through Day 42. Specifically, the Ur/Ue ratio, reflecting net elasticity and immediate recovery, and the Ur/Uf ratio, representing biological elasticity and overall skin distensibility, both demonstrated statistically significant increases after 42 days [7]. A moderate negative correlation was observed between patient age and baseline Ur/Uf values, indicating that older subjects tended to present lower net skin elasticity at baseline (Table S9). This observation is consistent with the literature and confirms that Ur/Uf is a relevant parameter to consider when evaluating skin biomechanical properties [7,54].

Broadly, no meaningful correlation was observed between patient age and treatment response. A subgroup analysis was performed according to age (i.e., <45 years versus ≥45 years). Among the 41 analyzed subjects, 17 were younger than 45 years and 24 were aged 45 years or older. At Day 14, the mean improvement from baseline was slightly higher in the ≥45 years group (*p* = 0.065) compared with the <45 years group (*p* = 0.053). However, at Day 42, the results were comparable between the two groups (*p* = 0.046 versus *p* = 0.048). The proportion of subjects showing improvement was also similar between groups at Day 42 (70.8% versus 70.6%). Overall, these findings suggest that age did not appear to be a major determinant of treatment response in this cohort.

The reported enhanced elasticity and firmness are crucial for restoring a youthful skin appearance, as they reflect the skin’s ability to withstand and recover from mechanical deformation [7,54]. These statistically significant increases in net elasticity (Ur/Ue) and biological elasticity (Ur/Uf) correlate directly with tangible, tactile improvements in tissue resilience.

The statistically significant increase in skin hydration, as quantified by Corneometer^®^ analysis, from a baseline of 49.39 ± 11.65 to 59.15 ± 10.22 at Day 14 and 63.52 ± 9.64 at Day 42 (*p*-value < 0.001), underscores HA and PN’s enduring capacity to enhance water retention within the dermis [49]. Skin hydration is a key biophysical parameter commonly used to evaluate general skin condition and barrier function. The water content of the stratum corneum (SC) is essential for proper corneocyte maturation and physiological desquamation [55]. Hydration measurements typically probe a superficial depth of approximately 10–20 μm, allowing selective assessment of the SC while minimizing interference from deeper skin structures. Due to its non-invasive measurement and quantitative output, SC hydration is widely used as an objective indicator of skin quality in dermatological and aesthetic studies [18,21,49,53,56]. The robust improvement in superficial SC hydration following a deep dermal injection highlights a vital cross-talk between skin layers. This sustained hydration is not only aesthetically beneficial, imparting a dewy and plump appearance, but also physiologically critical for maintaining the skin’s barrier function and overall health. Adequate hydration ensures optimal cellular function, facilitates nutrient delivery, and enhances the skin’s ability to withstand environmental stressors.

The high levels of investigator and subject satisfaction, reflected in 100% GAIS improvement at Day 0 and sustained high agreement rates in subjective questionnaires, underscore the comprehensive improvements in skin quality achieved with HYDRAGEL A2. The subjective assessments, which captured patient-reported outcomes, corroborated the objective instrumental measurements, highlighting the clinical relevance of the observed improvements. The mild and transient injection site reactions (ISRs), primarily observed immediately post-injection (Day 0) and spontaneously resolving by week 2 (Day 14), are consistent with the expected physiological inflammatory response to injectable hydrogels and are indicative of the body’s natural tissue integration process (Tables S7 and S8).

The absence of serious adverse events further supports the safety profile of the investigational product. Crucially, this favorable safety profile was consistently observed across a diverse demographic encompassing Fitzpatrick skin phototypes II through V. In aesthetic dermatology, treating patients with darker skin tones inherently carries a heightened risk of post-inflammatory hyperpigmentation (PIH) secondary to injection trauma [57]. The complete absence of reported pigmentary anomalies or sustained discoloration across these groups highlights the ‘color-blind’ safety of this biostimulatory approach. This broad tolerability may likely attributable to the anti-inflammatory properties of both PN and niacinamide, which can downregulate the acute inflammatory cascades that would otherwise trigger melanocyte hyperactivity in melanin-rich skin. The reported clinical use of HYDRAGEL A2 demonstrated a favorable safety and injectability profile, crucial for its adoption in commercial clinical practice. Furthermore, the product’s rheological properties, carefully optimized for dermal injection, facilitated smooth and controlled delivery, minimizing patient discomfort and ensuring precise placement within the targeted tissue layers. The injectability, assessed through ease of extrusion and uniform distribution, was well-regarded by investigators, contributing to a standardized and reproducible procedure (Table 2). Despite the presence of high-molecular-weight HA (1.8 MDa) and PNs (1.5 MDa), which naturally increase static viscosity and provide robust structural support, the application of mechanical stress during syringe extrusion causes the polymer chains to temporarily align. This allows the hydrogel to flow smoothly through fine-gauge needles or cannulas with minimal injector fatigue. This confirmed safety and ease of administration, coupled with the observed clinical efficacy, positions HYDRAGEL A2 as a reliable and well-tolerated option for facial rejuvenation. The rheological properties of the hydrogel, including its viscosity and elasticity, are carefully optimized to ensure predictable tissue integration, minimizing the risk of AEs and maximizing aesthetic outcomes.

#### 3.7.4. Outlook on the Temporality of the Clinical Results

The observed sustained improvements in skin quality with HYDRAGEL A2, extending through the Day 42 endpoint, suggest a promising longevity of the injection effects. However, a nuanced understanding of the dynamic interplay between the product’s components and the skin’s biological responses is essential for predicting long-term outcomes. The initial remodeling and hydrating effects of HA, observed immediately post-injection, are primarily due to its hygroscopic properties, creating a temporary increase in dermal support and hydration.

Thus, as HA is gradually metabolized by hyaluronidases and ROS, the immediate plumping effect is expected to diminish, typically within several weeks. Of note, the metabolism of HA is highly dynamic, as endogenous HA has a half-life of approximately 1 day in the dermis [17]. The incorporation of niacinamide may help protect HA from ROS-mediated degradation, while PNs contribute to increasing the viscosity of the matrix, thereby enhancing its structural stability and potentially prolonging its local transient persistence within the tissues [35,36].

In contrast, the pro-regenerative effects of PNs are anticipated to unfold over a more extended timeframe, contributing to more durable improvements. According to the literature, the initial phase, which may be occurring within the first few weeks, involves the activation of fibroblasts and the upregulation of growth factors such as VEGF, leading to improved local microcirculation which may facilitate the delivery of nutrients and oxygen, optimizing cellular function and accompanying tissue repair [20,22,27,35,37].

PNs may stimulate the production of type I and III collagen, which are essential for dermal structure and elasticity [20,22,27,35]. This process may be associated with a gradual increase in skin thickness and density, as observed in Dermascan^®^ imaging. Furthermore, the PNs’ ability to reduce oxidative stress contributes to a healthier dermal environment [20,22,44,46]. The sustained improvements in skin elasticity and firmness, as measured by using the Cutometer^®^, reflect these long-term structural changes.

Future longitudinal studies are crucial to determine the precise duration of these benefits and to assess the potential need for maintenance product administrations. These studies should incorporate serial imaging and biomechanical assessments to monitor the long-term changes in both the dermal structure and function. Additionally, investigating the potential for cumulative effects with repeated injections, including optimal injection intervals and dosages, will provide a more comprehensive understanding of HYDRAGEL A2’s long-term efficacy and its role in sustained facial rejuvenation. These studies should also investigate the potential for patient-specific reactions, and any long-term changes in skin quality.

### 3.8. Study Limitations and Future Perspectives

While this study successfully demonstrated that HYDRAGEL A2 provides a well-tolerated approach associated with improvements in skin quality, several limitations warrant acknowledgment and provide avenues for future research. The single-center design and relatively small sample size, while adequate for an initial safety and efficacy assessment, inherently limit the generalizability of the findings to broader and more diverse populations. Furthermore, the exclusive enrollment of female subjects (i.e., 100% of the study population) represents a notable limitation, as inherent variations in skin thickness, aging patterns, and physiological responses to biopolymers exist between biological sexes. The open-label, self-controlled design also introduces inherent investigator and subject bias, particularly regarding subjective metrics like the GAIS. Environmental and seasonal confounders occurring over the 6-week period could also potentially influence sensitive biophysical parameters such as stratum corneum hydration. Overall, variations in skin types, environmental exposures, and genetic backgrounds across different populations could influence treatment outcomes, necessitating multi-center studies with larger, more heterogeneous cohorts.

The absence of a comparator group, such as a placebo or active control, restricts the ability to directly compare HYDRAGEL A2’s performance to other available injectable devices or to isolate the specific contributions of each ingredient. Future research should incorporate randomized controlled trials (RCTs) with active comparators to rigorously assess HYDRAGEL A2’s superiority or equivalence to existing aesthetic interventions. This would allow for a more nuanced understanding of the product’s clinical positioning and optimize its selection based on patient needs. Additionally, future studies could include a dedicated sub-cohort in which injections are performed in a less visible anatomical area (e.g., post-auricular region), followed by skin biopsies for histological analysis, to provide deeper insight into ECM changes and better characterize tissue-level effects.

Long-term follow-up studies are crucial to assess the durability of the observed improvements and to evaluate any potential long-term adverse effects. While the current study demonstrated sustained benefits through Day 42, the temporal dynamics of dermal remodeling and in vivo ingredient degradation necessitate extended monitoring periods. These studies should include serial imaging, biomechanical assessments, and patient-reported outcomes to track changes in skin structure, function, and aesthetic appearance over time. Additionally, investigating the potential for cumulative effects with repeated injections and determining optimal maintenance injection schedules are essential for long-term clinical management. From a translational perspective, it would be of interest to investigate the potential role of HYDRAGEL A2 as a preparatory treatment prior to the administration of long-acting biostimulatory injectables. The latter notably comprise products such as Sculptra^®^ (Galderma S.A., Uppsala, Sweden) and Julaïne™ of Sweden (Nordberg Medical AB, Stockholm, Sweden), primarily composed of poly-L-lactic acid, as well as Radiesse^®^ (Merz Aesthetics (Suisse) SA, Allschwil, Switzerland), Hydroxyal (Louna Aesthetics, Poisy, France), or Novuma^®^ (Burgeon Biotechnology, Ankara, Turkey), based on calcium hydroxylapatite (CaHA). In this context, locally preparing the dermal environment with a pro-regenerative hydrogel may enhance the biological responsiveness of the tissue and potentially improve the long-term outcomes of subsequent biostimulatory treatments. Of note, the concept of ‘tissue priming’ represents a critical paradigm shift. Because long-acting biostimulators like poly-L-lactic acid (PLLA) or CaHA rely on a robust host macrophage and fibroblast response for collagen induction, injecting them into senescent, dehydrated, or chronically inflamed tissue can yield suboptimal or unpredictable results. By pre-conditioning the ECM with the anti-inflammatory properties of niacinamide and the regenerative cues of PNs, clinicians could theoretically convert a sluggish cellular environment into a highly responsive one, making this hydrogel an essential foundational step in comprehensive restorative protocols. Future studies investigating such sequential injection strategies could provide valuable insights into optimizing regenerative aesthetic protocols.

Additionally, future studies should explore the potential for personalized injection approaches, considering individual patient characteristics such as age, skin type, and baseline dermal parameters. Investigating the influence of these factors on injection response could lead to tailored injection protocols, maximizing patient outcomes and satisfaction. Finally, research focusing on the optimization of the hydrogel’s rheological properties and injection techniques would contribute to improved product delivery and patient comfort, further enhancing the clinical utility of HYDRAGEL A2.

## 4. Conclusions

Generally, the present first clinical investigation demonstrated that HYDRAGEL A2, an injectable medical device containing HA, PNs, and niacinamide, is a well-tolerated approach associated with improvements in facial skin quality in healthy subjects aged 30 to 59 years. The device demonstrated a favorable safety and tolerability profile, with only mild and transient ISRs, such as redness, pain, hardening, swelling, bumps, bruising, itching, and discoloration, primarily observed immediately post-injection (D0) and spontaneously resolving by week 2 (D14). These reactions were within the expected range, and no subjects reported allergic reactions. The complete absence of allergic hypersensitivity or serious adverse events over the 6-week observation period serves as a critical biological validation of the complex’s macromolecular purity. The rigorous extraction and purification processes required to isolate high-molecular-weight PNs successfully eliminated immunogenic proteins, yielding a highly biocompatible matrix.

The efficacy of HYDRAGEL A2 was evidenced by important and sustained improvements in skin texture, elasticity, hydration, density, and thickness. On D0 post-injection, 100% of the 43 subjects exhibited aesthetic improvements, as per the GAIS. The investigational device not only achieved immediate effectiveness on Day 0, but also maintained high improvement rates on Day 14 and Day 42. Specifically, Antera 3D^®^ analysis revealed significant reductions in skin roughness (Ra and Rq) at both D14 and D42, indicating enhanced skin smoothness. Cutometer^®^ measurements demonstrated significant improvements in skin elasticity and firmness, with Ur/Ue and Ur/Uf parameters showing sustained benefits through D42. Corneometer^®^ analysis showed a statistically significant increase in skin hydration at D14 (59.15 ± 10.22) and D42 (63.52 ± 9.64) compared to baseline (49.39 ± 11.65, *p* < 0.001). Dermascan^®^ imaging confirmed increased skin thickness and density, indicating structural improvements, with parameters such as segmented area, total intensity, and thickness showcasing dynamic responses and reduced irregularities.

Both investigators and subjects reported high levels of satisfaction with the intervention outcomes. Subjective questionnaires also reflected a growing satisfaction with the administered device over time, indicating perceived improvements in individual facial skin attributes and a reduction in fine lines. The observed increase in the percentage of subjects who “Agree” or “Totally agree” about improvements in their skin condition reflects growing overall satisfaction with the intervention over time.

The objective instrumental assessments, combined with high investigator and subject satisfaction, support the use of HYDRAGEL A2 as a valuable option for individuals seeking non-surgical, minimally invasive interventions aiming to enhance their skin quality. Clinically, this positions the investigational device within a highly specialized niche. It effectively bridges the gap between traditional crosslinked volumizers and standalone biostimulators, which often suffer from a prolonged latency period before results are visible. By delivering immediate aesthetic improvement without compromising the long-term biological safety profile, this hydrogel offers practitioners a reliable tool for wide-field skin redensification.

Overall, the HYDRAGEL A2 device shows promising and sustained improvements in various skin quality attributes, reinforcing its potential for effective modulation of facial skin aging. Further research, including long-term follow-up studies and comparative analyses with other injectable devices, would be beneficial to further elucidate the full clinical potential of HYDRAGEL A2. Specifically, future clinical investigations should formally evaluate the role of this biopolymer complex in ‘tissue priming’ protocols. As the aesthetic industry moves toward comprehensive, multi-modal rejuvenation, establishing an optimal biological canvas is essential. By reversing superficial dermal senescence, downregulating chronic inflammaging, and restoring the ECM’s hydration and elasticity, pre-treating the skin with this PN/HA/niacinamide triad could theoretically amplify the clinical efficacy and safety of subsequent, more aggressive interventions, such as energy-based devices or deep structural biostimulators. However, the current study provides preliminary clinical evidence supporting its potential use in clinical practice for improving skin quality and addressing the visible signs of facial aging.

## Figures and Tables

**Table 1:** Study demographic parameters.

Parameters		Study Population (n = 42)	
**Age**	Mean	45	
	Median	43	
	Minimum	30	
	Maximum	59	
	SD	7	
	SEM	1	
	95% CI	2	
**Gender**	Female	42	100.00%
	Male	0	0%
	**Total**	**42**	**100.00%**
**Ethnicity**	Caucasian	8	19.05%
	Mixed race	17	40.48%
	Indian	8	19.05%
	Asian	3	7.14%
	African	6	14.28%
	**Total**	**42**	**100.00%**
**Phototype**	I	0	0%
	II	1	2.38%
	III	11	26.19%
	IV	17	40.48%
	V	13	30.95%
	VI	0	0%
	**Total**	**42**	**100.00%**

**Table 2:** Study flowchart.

Visit	Screening Visit	Visit 1	Visit 2	Visit 3	Visit 4
Day	D-30	D-3–D0	D0	W2 (±3 Days)	W6 (±7 Days)
Informed consent	X	-	-	-	-
Clinical examination to check inclusion and non-inclusion criteria	X	-	-	-	-
Medical history and previous treatment(s)	X	-	-	-	-
Inclusion/Non-inclusion decision	X	-	-	-	-
Urinary pregnancy test (UPT)	-	X	-	-	-
Injection	-	-	X	-	-
GAIS (Global Aesthetic Improvement Scale) by investigator and by subjects	-	-	X *	X	X
ISR (Injection Site Reaction) by the investigator	-	-	X *	X *	X
ISR (Injection Site Reaction) by the subject (each day)	-	-	X	X	X
Subjective evaluation questionnaire by injector	-	-	X *	-	-
2D Photographs: Whole face and profile Small area on cheek	-	X X	-	X X	X X
Image acquisition for skin texture analysis with the Antera 3D^®^	-	X	-	X	X
Skin elasticity measurement with the Cutometer^®^	-	X	-	X	X
Echography (for density and thickness) using the Dermascan^®^	-	X	-	X	X
Skin hydration measurement using the Corneometer^®^	-	X	-	X	X
Adherence control, record of adverse events, concomitant treatments	-	-	X	X	X
Subjective evaluation questionnaire by the patient	-	-	-	X	X

* After injection.

**Table 3:** Count and percentage (n [%]) of subjects for the subjective questionnaire.

Questions	Timepoints	1: Totally Disagree	2: Disagree	3: Neither Agree Nor Disagree	4: Agree	5: Totally Agree
Improvement in skin firmness	D14 D42	0 (0.0%) 0 (0.0%)	0 (0.0%) 0 (0.0%)	8 (19.0%) 1 (2.4%)	23 (54.8%) 24 (57.1%)	11 (26.2%) 17 (40.5%)
Improvement of skin elasticity/pliability	D14 D42	0 (0.0%) 0 (0.0%)	0 (0.0%) 0 (0.0%)	8 (19.0%) 2 (4.8%)	25 (59.5%) 23 (54.8%)	9 (21.4%) 17 (40.5%)
Improvement in skin laxity	D14 D42	0 (0.0%) 0 (0.0%)	0 (0.0%) 0 (0.0%)	13 (31.0%) 3 (7.1%)	23 (54.8%) 22 (52.4%)	6 (14.3%) 17 (40.5%)
Improvement in skin hydration	D14 D42	0 (0.0%) 0 (0.0%)	0 (0.0%) 0 (0.0%)	5 (11.9%) 0 (0.0%)	29 (69.0%) 25 (59.5%)	8 (19.0%) 17 (40.5%)
Lifting effect	D14 D42	0 (0.0%) 0 (0.0%)	1 (2.4%) 0 (0.0%)	6 (14.3%) 2 (4.8%)	28 (66.7%) 26 (61.9%)	7 (16.7%) 14 (33.3%)
Decrease of skin fine lines	D14 D42	0 (0.0%) 0 (0.0%)	2 (4.8%) 0 (0.0%)	11 (26.2%) 6 (14.3%)	25 (59.5%) 21 (50.0%)	4 (9.5%) 15 (35.7%)
Improvement of the aspect of skin pores	D14 D42	0 (0.0%) 0 (0.0%)	0 (0.0%) 0 (0.0%)	7 (16.7%) 3 (7.1%)	26 (61.9%) 24 (57.1%)	9 (21.4%) 15 (35.7%)
I am satisfied with the result obtained	D14 D42	0 (0.0%) 0 (0.0%)	0 (0.0%) 0 (0.0%)	8 (19.0%) 2 (4.8%)	23 (54.8%) 21 (50.0%)	11 (26.2%) 19 (45.2%)

**Table 4:** Count and percentage (i.e., n [%]) of subject cases for ease of product administration, based on the injector’s assessments.

Parameter	Very Satisfied	Satisfied	Neither Satisfied Nor Dissatisfied	Dissatisfied	Very Dissatisfied
Ease of injection	37 (86.0%)	5 (11.6%)	1 (2.3%)	0 (0.0%)	0 (0.0%)
Immediate result	13 (30.2%)	30 (69.8%)	0 (0.0%)	0 (0.0%)	0 (0.0%)

## Data Availability

The data presented in this study are openly available within the article files.

## Supplementary Materials

The following supporting information can be downloaded at: https://www.mdpi.com/article/10.3390/jfb17050254/s1, Figure S1: Anatomical zones of injection; Figure S2: Rheological attributes; Figure S3: Images of the left cheek from four selected study patients; Table S1: Nomenclature definitions; Tables S2–S9: Detailed numerical results and statistical analyses of the results of the clinical study.

## Abbreviations

AE	adverse event
CRO	contract research organization
DNA	deoxyribonucleic acid
ECM	extracellular matrix
EMA	European Medicines Agency
FDA	Food and Drug Administration
G′	storage modulus
G″	loss modulus
GAIS	global aesthetic improvement scale
GCP	good clinical practices
HA	hyaluronic acid
IGAIS	investigator global aesthetic improvement scale
ISR	injection site reactions
mRNA	messenger ribonucleic acid
NA	non-applicable
ns	non-significant
Pa	Pascals
Pa·s	Pascal seconds
ROS	reactive oxygen species
s	second
SAE	serious adverse events
SGAIS	subject global aesthetic improvement scale
USA	United States of America
