# Supplementary material for: An Injectable Biopolymer Hydrogel Complex (PN/HA/B3) for Facial Skin Redensification and General Rejuvenation: Clinical Report on Device Safety and Efficacy
Source: J Funct Biomater. 2026 May 20;17(5):254. doi: 10.3390/jfb17050254 (PMC13208250; doi:10.3390/jfb17050254)
Supplement: Supplementary file 1 [file jfb-17-00254-s001.zip › jfb-4276992 Supplementary.pdf]

Supplementary Materials:

An Injectable Biopolymer Hydrogel Complex (PN/HA/B3) for Facial Skin Redensification and General Rejuvenation: Clinical Report on Device Safety and Efficacy

Alexandre Porcello, Kelly Lourenço, Cíntia Marques, Wassim Raffoul, Marco Cerrano, Lee Ann Applegate and Alexis E. Laurent *

1. Supplementary Figures

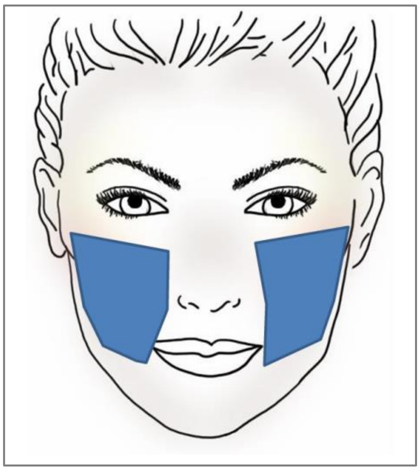

Figure S1. Zones of the facial skin which were targeted for product administration.

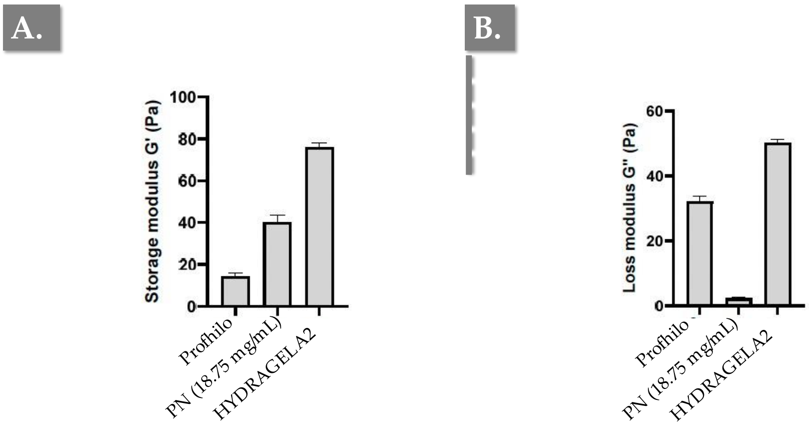

Figure S2. Results of rheological attributes of Profhilo®, a formulation of PN at 18.75 mg/mL and HYDRAGEL A2 after extrusion through a 25G canula at 22°C and 1 Hz. the samples were analyzed in oscillatory rheology at 22°C with a frequency of 1 Hz. Data expressed as G′ storage modulus and G′′ loss modulus are presented. Measurements were performed in triplicate and standard deviations were reported as error bars around mean

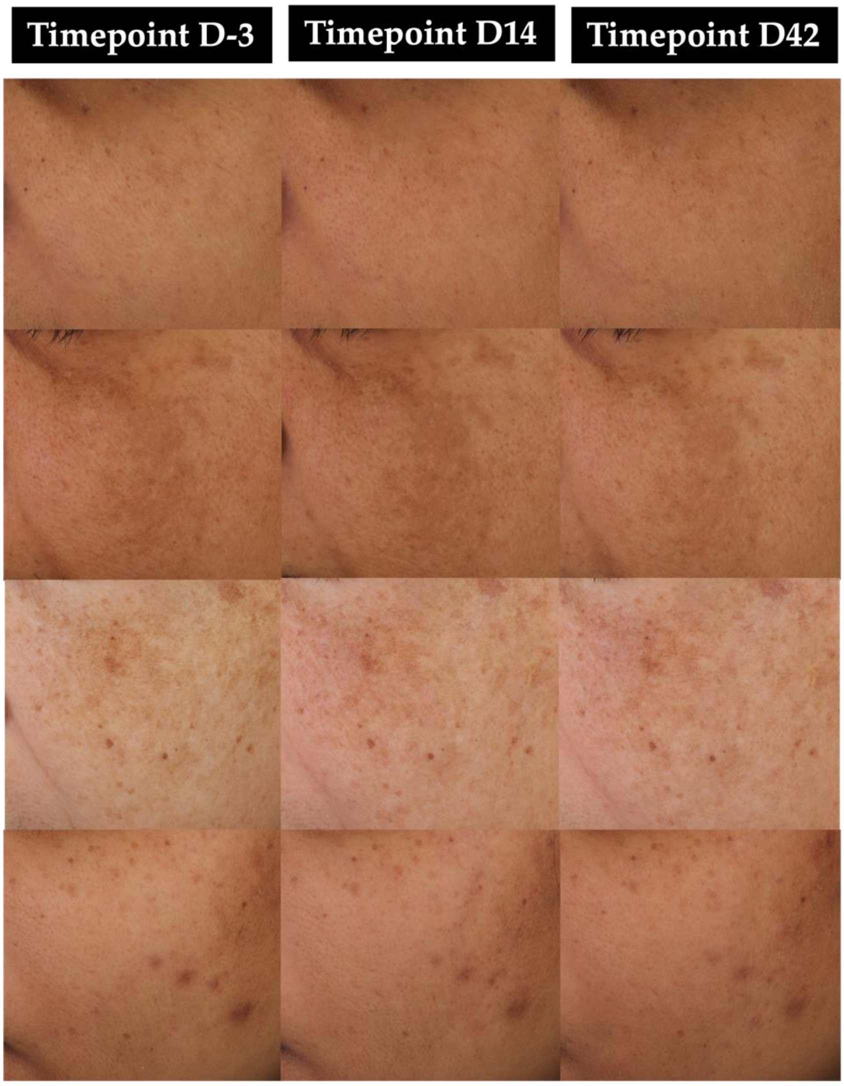

Figure S3. Images of the left cheek from four selected study patients.

2. Supplementary Tables

Table S1. Description of the significance levels for global aesthetic improvement scores.

Rating		Description
1	Very much improved	Optimal cosmetic results in this subject
2	Much improved	Marked improvement in appearance form the initial condition, but not completely optimal for this subject
3	Improved	Obvious improvement in appearance from initial condition, but a re-treatment is indicated
4	No change	The appearance is essentially the same as the original condition
5	Worse	The appearance is worse than the original condition

Table S2. Count and percentage (n[%]) of subjects for GAIS scores, by evaluator and category. Statistical analysis was performed with a binomial test of proportion versus 0.4. GAIS, global aesthetic improvement scale.

Evaluator	Timepoint	n (%)		95% CI		*p*-value
		Improved	Not improved	Lower	Upper	(Significance)
By investigator	D0 after injection	43 (100.0%)	0 (0.0%)	93.27	100.00	**<0.001 (S)**
	D14	42 (100.0%)	0 (0.0%)	93.12	100.00	**<0.001 (S)**
	D42	42 (100.0%)	0 (0.0%)	93.12	100.00	**<0.001 (S)**
By subject	D0 after injection	43 (100.0%)	0 (0.0%)	93.27	100.00	**<0.001 (S)**
	D14	41 (97.6%)	1 (2.4%)	89.20	100.00	**<0.001 (S)**
	D42	40 (95.2%)	2 (4.8%)	85.76	100.00	**<0.001 (S)**
Combined	D0 after injection	43 (100.0%)	0 (0.0%)	93.27	100.00	**<0.001 (S)**
	D14	41 (97.6%)	1 (2.4%)	89.20	100.00	**<0.001 (S)**
	D42	40 (95.2%)	2 (4.8%)	85.76	100.00	**<0.001 (S)**

Table S3. Descriptive statistics and evolution over time for skin texture parameters analyzed with the Antera 3D device. When compared to D–3, a significant improvement was noted for all parameters of interest derived from image processing, characterizing texture and roughness.

Parameter	Timepoint	n	Mean	Median	SD	Minimum	Maximum	% Variation	*p*-Value (Significance) Statistical test used
Texture Score	D-3	42	41.12	38.25	13.56	20.00	79.50		
Texture Score	D14	42	35.79	34.00	12.15	18.50	78.00		
Texture Score	D42	42	37.61	35.00	12.88	19.50	80.50		
Texture Score	D14 – D–3	42	–5.33	–5.50	3.87	–21.50	0.00	–12.97	**<0.001 (S) Wilcoxon**
Texture Score	D42 – D–3	42	–3.51	–3.00	3.08	–12.00	1.00	–8.54	**<0.001 (S) Wilcoxon**
Roughness Ra (µm)	D–3	42	9.07	8.53	2.15	6.05	15.76		
Roughness Ra (µm)	D14	42	8.26	7.90	1.88	5.80	15.53		
Roughness Ra (µm)	D42	42	8.54	8.10	2.04	5.93	16.06		
Roughness Ra (µm)	D14 – D–3	42	–0.81	–0.72	0.65	–3.68	0.06	–8.93	**<0.001 (S) Wilcoxon**
Roughness Ra (µm)	D42 – D–3	42	–0.52	–0.46	0.50	–2.13	0.30	–5.78	**<0.001 (S) Wilcoxon**
Roughness Rq (µm)	D–3	42	11.61	10.86	2.73	7.92	19.78		
Roughness Rq (µm)	D14	42	10.60	10.11	2.38	7.51	19.72		
Roughness Rq (µm)	D42	42	10.96	10.46	2.57	7.71	20.23		
Roughness Rq (µm)	D14 – D–3	42	–1.02	–0.91	0.87	–4.69	0.11	–8.75	**<0.001 (S) Wilcoxon**
Roughness Rq (µm)	D42 – D–3	42	–0.65	–0.53	0.66	–2.68	0.45	–5.58	**<0.001 (S) Wilcoxon**
Maximum height (mm)	D–3	42	0.10	0.10	0.02	0.06	0.17		
Maximum height (mm)	D14	42	0.10	0.09	0.02	0.06	0.17		
Maximum height (mm)	D42	42	0.10	0.09	0.02	0.06	0.15		
Maximum height (mm)	D14 – D–3	42	0.00	–0.01	0.01	–0.03	0.04	–4.62	**0.033 (S) Paired t-test**
Maximum height (mm)	D42 – D–3	42	0.00	0.00	0.01	–0.03	0.02	–3.77	**0.025 (S) Paired t-test**

Table S4. Descriptive statistics and evolution over time for skin texture parameters analyzed with the DermaScan device.

Parameter	Timepoint	n	Mean	Median	SD	Minimum	Maximum	% Variation	*p*-Value (Significance) Statistical test used
Segmented Area (mm^2)	D–3	42	7.24	6.91	1.49	5.41	12.07		
	D14	42	5.58	5.40	1.07	4.10	9.37		
	D42	42	6.12	5.79	1.22	4.69	10.70		
	D14 – D–3	42	–1.66	–1.38	0.80	–5.19	–0.67	–22.96	**<0.001 (S) Wilcoxon**
	D42 – D–3	42	–1.12	–0.91	0.65	–3.80	–0.44	–15.46	**<0.001 (S) Wilcoxon**
Total Intensity (%)	D–3	42	21.30	21.42	3.67	13.49	29.66		
	D14	42	23.12	23.31	5.08	12.12	34.31		
	D42	42	19.62	19.33	4.03	9.97	28.50		
	D14 – D–3	42	1.82	2.19	4.36	–8.88	9.52	8.55	**0.010 (S) Paired t-test**
	D42 – D–3	42	–1.68	–2.24	4.52	–9.50	8.65	–7.90	**0.020 (S) Paired t-test**
Thickness	D–3	42	1.09	1.09	0.04	1.03	1.19		
	D14	42	1.48	1.49	0.08	1.18	1.60		
	D42	42	1.29	1.29	0.05	1.09	1.37		
	D14 – D–3	42	0.39	0.40	0.09	0.16	0.53	35.44	**<0.001 (S) Paired t-test**
	D42 – D–3	42	0.19	0.19	0.06	0.07	0.31	17.83	**<0.001 (S) Paired t-test**

Table S5. Descriptive statistics and evolution over time for skin texture parameters analyzed with the Cutometer device. A general significant increase was noted over time for all parameters assessed (i.e., whether absolute or relative). The change was more important for the relative parameters (e.g., Ua/Uf, Ur/Ue, and Ur/Uf), which is suggestive of improved skin features characterizing firmness and elasticity.

Parameter	Timepoint	n	Mean	Median	SD	Minimum	Maximum	% Variation	*p*-Value (Significance) Statistical test used
Ue	D–3	42	0.25	0.25	0.05	0.15	0.35		
	D14	42	0.27	0.26	0.05	0.16	0.36		
	D42	42	0.25	0.24	0.05	0.13	0.36		
	D14 – D–3	42	0.02	0.01	0.05	–0.09	0.12	7.06	**0.019 (S) Paired t-test**
	D42 – D–3	42	0.00	0.00	0.05	–0.12	0.11	0.97	0.763 (NS) Paired t-test
Uf	D–3	42	0.32	0.32	0.05	0.20	0.41		
	D14	42	0.34	0.34	0.05	0.24	0.45		
	D42	42	0.33	0.32	0.06	0.19	0.46		
	D14 – D–3	42	0.02	0.02	0.05	–0.08	0.13	7.02	**0.003 (S) Paired t-test**
	D42 – D–3	42	0.01	0.00	0.06	–0.11	0.18	1.77	0.525 (NS) Paired t-test
Ua/Uf	D–3	42	0.74	0.74	0.09	0.56	0.92		
	D14	42	0.78	0.79	0.08	0.60	0.97		
	D42	42	0.76	0.74	0.10	0.59	0.92		
	D14 – D–3	42	0.05	0.05	0.09	–0.19	0.18	6.30	**0.002 (S) Paired t-test**
	D42 – D–3	42	0.02	0.02	0.09	–0.21	0.19	2.67	0.180 (NS) Paired t-test
Ur/Ue	D–3	42	0.60	0.61	0.12	0.38	0.83		
	D14	42	0.68	0.68	0.13	0.42	0.90		
	D42	42	0.67	0.65	0.13	0.47	0.93		
	D14 – D–3	42	0.08	0.08	0.06	–0.12	0.22	13.47	**<0.001 (S) Paired t-test**
	D42 – D–3	42	0.07	0.08	0.09	–0.16	0.24	11.40	**<0.001 (S) Paired t-test**
Ur/Uf	D–3	42	0.47	0.47	0.10	0.28	0.66		
	D14	42	0.53	0.55	0.10	0.32	0.69		
	D42	42	0.51	0.51	0.11	0.33	0.73		
	D14 – D–3	42	0.06	0.06	0.05	–0.08	0.17	12.63	**<0.001 (S) Paired t-test**
	D42 – D–3	42	0.05	0.06	0.08	–0.19	0.19	9.86	**<0.001 (S) Paired t-test**

Table S6. Descriptive statistics and evolution over time for skin hydration parameters analyzed with the Corneometer device. A significant increase in the hydration level was observed at all timepoints when compared to D–3.

Timepoint	n	Mean	Median	SD	Minimum	Maximum	% Variation	*p*-Value (Significance) Statistical test used
D–3	42	49.39	49.02	11.65	31.00	69.77		
D14	42	59.15	60.13	10.22	39.92	75.83		
D42	42	63.52	64.51	9.64	45.37	82.57		
D14 – D–3	42	9.76	7.11	7.35	1.22	40.80	19.76	**<0.001 (S) Wilcoxon**
D42 – D–3	42	14.13	12.84	9.49	-5.27	47.65	28.60	**<0.001 (S) Wilcoxon**

Table S7. Count and percentage (n [%]) of subjects for ISR, as evaluated by the investigator.

Parameter	Sides	Timepoint	0: None	1: Light	2: Moderate	3: Severe	Absence (0)	Presence (1, 2, 3)
Redness	Right	D0 (after injection)	23 (53.5%)	18 (41.9%)	2 (4.7%)	0 (0.0%)	23 (53.5%)	20 (46.5%)
		D14	42 (100.0%)	0 (0.0%)	0 (0.0%)	0 (0.0%)	42 (100.0%)	0 (0.0%)
		D42	42 (100.0%)	0 (0.0%)	0 (0.0%)	0 (0.0%)	42 (100.0%)	0 (0.0%)
	Left	D0 (after injection)	21 (48.8%)	20 (46.5%)	2 (4.7%)	0 (0.0%)	21 (48.8%)	22 (51.2%)
		D14	42 (100.0%)	0 (0.0%)	0 (0.0%)	0 (0.0%)	42 (100.0%)	0 (0.0%)
		D42	42 (100.0%)	0 (0.0%)	0 (0.0%)	0 (0.0%)	42 (100.0%)	0 (0.0%)
Pain/ Sensitivity	Right	D0 (after injection)	13 (30.2%)	24 (55.8%)	6 (14.0%)	0 (0.0%)	13 (30.2%)	30 (69.8%)
		D14	42 (100.0%)	0 (0.0%)	0 (0.0%)	0 (0.0%)	42 (100.0%)	0 (0.0%)
		D42	42 (100.0%)	0 (0.0%)	0 (0.0%)	0 (0.0%)	42 (100.0%)	0 (0.0%)
	Left	D0 (after injection)	14 (32.6%)	23 (53.5%)	6 (14.0%)	0 (0.0%)	14 (32.6%)	29 (67.4%)
		D14	42 (100.0%)	0 (0.0%)	0 (0.0%)	0 (0.0%)	42 (100.0%)	0 (0.0%)
		D42	42 (100.0%)	0 (0.0%)	0 (0.0%)	0 (0.0%)	42 (100.0%)	0 (0.0%)
Hardening/ Firmness	Right	D0 (after injection)	0 (0.0%)	43 (100.0%)	0 (0.0%)	0 (0.0%)	0 (0.0%)	43 (100.0%)
		D14	42 (100.0%)	0 (0.0%)	0 (0.0%)	0 (0.0%)	42 (100.0%)	0 (0.0%)
		D42	42 (100.0%)	0 (0.0%)	0 (0.0%)	0 (0.0%)	42 (100.0%)	0 (0.0%)
	Left	D0 (after injection)	0 (0.0%)	43 (100.0%)	0 (0.0%)	0 (0.0%)	0 (0.0%)	43 (100.0%)
		D14	42 (100.0%)	0 (0.0%)	0 (0.0%)	0 (0.0%)	42 (100.0%)	0 (0.0%)
		D42	42 (100.0%)	0 (0.0%)	0 (0.0%)	0 (0.0%)	42 (100.0%)	0 (0.0%)
Swelling	Right	D0 (after injection)	1 (2.3%)	42 (97.7%)	0 (0.0%)	0 (0.0%)	1 (2.3%)	42 (97.7%)
		D14	42 (100.0%)	0 (0.0%)	0 (0.0%)	0 (0.0%)	42 (100.0%)	0 (0.0%)
		D42	42 (100.0%)	0 (0.0%)	0 (0.0%)	0 (0.0%)	42 (100.0%)	0 (0.0%)
	Left	D0 (after injection)	1 (2.3%)	42 (97.7%)	0 (0.0%)	0 (0.0%)	1 (2.3%)	42 (97.7%)
		D14	42 (100.0%)	0 (0.0%)	0 (0.0%)	0 (0.0%)	42 (100.0%)	0 (0.0%)
		D42	42 (100.0%)	0 (0.0%)	0 (0.0%)	0 (0.0%)	42 (100.0%)	0 (0.0%)
Bumps	Right	D0 (after injection)	7 (16.3%)	36 (83.7%)	0 (0.0%)	0 (0.0%)	7 (16.3%)	36 (83.7%)
		D14	42 (100.0%)	0 (0.0%)	0 (0.0%)	0 (0.0%)	42 (100.0%)	0 (0.0%)
		D42	42 (100.0%)	0 (0.0%)	0 (0.0%)	0 (0.0%)	42 (100.0%)	0 (0.0%)
	Left	D0 (after injection)	7 (16.3%)	36 (83.7%)	0 (0.0%)	0 (0.0%)	7 (16.3%)	36 (83.7%)
		D14	42 (100.0%)	0 (0.0%)	0 (0.0%)	0 (0.0%)	42 (100.0%)	0 (0.0%)
		D42	42 (100.0%)	0 (0.0%)	0 (0.0%)	0 (0.0%)	42 (100.0%)	0 (0.0%)
Bruising	Right	D0 (after injection)	43 (100.0%)	0 (0.0%)	0 (0.0%)	0 (0.0%)	43 (100.0%)	0 (0.0%)
		D14	42 (100.0%)	0 (0.0%)	0 (0.0%)	0 (0.0%)	42 (100.0%)	0 (0.0%)
		D42	42 (100.0%)	0 (0.0%)	0 (0.0%)	0 (0.0%)	42 (100.0%)	0 (0.0%)
	Left	D0 (after injection)	43 (100.0%)	0 (0.0%)	0 (0.0%)	0 (0.0%)	43 (100.0%)	0 (0.0%)
		D14	42 (100.0%)	0 (0.0%)	0 (0.0%)	0 (0.0%)	42 (100.0%)	0 (0.0%)
		D42	42 (100.0%)	0 (0.0%)	0 (0.0%)	0 (0.0%)	42 (100.0%)	0 (0.0%)
Itching	Right	D0 (after injection)	43 (100.0%)	0 (0.0%)	0 (0.0%)	0 (0.0%)	43 (100.0%)	0 (0.0%)
		D14	42 (100.0%)	0 (0.0%)	0 (0.0%)	0 (0.0%)	42 (100.0%)	0 (0.0%)
		D42	42 (100.0%)	0 (0.0%)	0 (0.0%)	0 (0.0%)	42 (100.0%)	0 (0.0%)
	Left	D0 (after injection)	43 (100.0%)	0 (0.0%)	0 (0.0%)	0 (0.0%)	43 (100.0%)	0 (0.0%)
		D14	42 (100.0%)	0 (0.0%)	0 (0.0%)	0 (0.0%)	42 (100.0%)	0 (0.0%)
		D42	42 (100.0%)	0 (0.0%)	0 (0.0%)	0 (0.0%)	42 (100.0%)	0 (0.0%)
Discoloration	Right	D0 (after injection)	43 (100.0%)	0 (0.0%)	0 (0.0%)	0 (0.0%)	43 (100.0%)	0 (0.0%)
		D14	42 (100.0%)	0 (0.0%)	0 (0.0%)	0 (0.0%)	42 (100.0%)	0 (0.0%)
		D42	42 (100.0%)	0 (0.0%)	0 (0.0%)	0 (0.0%)	42 (100.0%)	0 (0.0%)
	Left	D0 (after injection)	43 (100.0%)	0 (0.0%)	0 (0.0%)	0 (0.0%)	43 (100.0%)	0 (0.0%)
		D14	42 (100.0%)	0 (0.0%)	0 (0.0%)	0 (0.0%)	42 (100.0%)	0 (0.0%)
		D42	42 (100.0%)	0 (0.0%)	0 (0.0%)	0 (0.0%)	42 (100.0%)	0 (0.0%)

Table S8. Count and percentage (n [%]) of subjects for ISR (maximum severity over the period of observation), as reported by subjects.

Parameter	Side	0: None	1: Light	2: Moderate	3: Severe
Redness	Right	9 (21.4%)	22 (52.4%)	10 (23.8%)	1 (2.4%)
	Left	12 (28.6%)	26 (61.9%)	4 (9.5%)	0 (0.0%)
Pain/ Sensitivity	Right	8 (19.0%)	25 (59.5%)	9 (21.4%)	0 (0.0%)
	Left	8 (19.0%)	27 (64.3%)	7 (16.7%)	0 (0.0%)
Hardening/ firmness	Right	4 (9.5%)	22 (52.4%)	15 (35.7%)	1 (2.4%)
	Left	5 (11.9%)	22 (52.4%)	15 (35.7%)	0 (0.0%)
Swelling	Right	4 (9.5%)	18 (42.9%)	20 (47.6%)	0 (0.0%)
	Left	6 (14.3%)	21 (50.0%)	15 (35.7%)	0 (0.0%)
Bumps	Right	9 (21.4%)	21 (50.0%)	12 (28.6%)	0 (0.0%)
	Left	11 (26.2%)	21 (50.0%)	10 (23.8%)	0 (0.0%)
Blue	Right	26 (61.9%)	10 (23.8%)	4 (9.5%)	2 (4.8%)
	Left	27 (64.3%)	10 (23.8%)	5 (11.9%)	0 (0.0%)
Itching	Right	38 (90.5%)	4 (9.5%)	0 (0.0%)	0 (0.0%)
	Left	38 (90.5%)	2 (4.8%)	1 (2.4%)	1 (2.4%)
Discoloration	Right	36 (85.7%)	5 (11.9%)	1 (2.4%)	0 (0.0%)
	Left	36 (85.7%)	6 (14.3%)	0 (0.0%)	0 (0.0%)

Table S9. Subjects characteristics with associated Ur/Ue, and Ur/Uf values. *: values not included in data analysis; NA: Not applicable; F: Femal subject; C: Caucasian M: Mixed race I: Indian Af: African

					Ur/Ue					Ur/Uf				
**Subject ID.**	**Age**	**Gender**	**Phototype**	**Ethnicity**	**D-3**	**D14**	**D42**	**D14-D-3**	**D42-D-3**	**D-3**	**D14**	**D42**	**D14-D-3**	**D42-D-3**
CIDP-MRU-0001	40	F	V	M	0.66	0.76	0.69	0.11	0.03	0.51	0.58	0.53	0.07	0.03
CIDP-MRU-0002	35	F	V	Af	0.67	0.70	0.69	0.02	0.02	0.51	0.56	0.51	0.06	0.01
CIDP-MRU-0003	38	F	V	Af	0.76	0.89	0.60	0.12	-0.16	0.61	0.58	0.42	-0.02	-0.19
CIDP-MRU-0004	56	F	IV	M	0.42	0.52	0.47	0.09	0.05	0.31	0.40	0.33	0.09	0.01
CIDP-MRU-0005	48	F	V	Af	0.39	0.57	0.58	0.17	0.19	0.28	0.39	0.38	0.11	0.10
CIDP-MRU-0006	36	F	IV	A	0.68	0.84	0.58	0.16	-0.10	0.55	0.61	0.47	0.06	-0.08
CIDP-MRU-0007	48	F	IV	I	0.44	0.56	0.50	0.12	0.06	0.31	0.44	0.39	0.12	0.07
CIDP-MRU-0009	36	F	V	M	0.76	0.90	0.71	0.15	-0.04	0.56	0.68	0.55	0.12	-0.02
CIDP-MRU-0010	43	F	IV	I	0.76	0.79	0.81	0.03	0.05	0.54	0.62	0.65	0.08	0.11
CIDP-MRU-0011	56	F	V	I	0.54	0.63	0.73	0.08	0.18	0.43	0.50	0.56	0.07	0.12
CIDP-MRU-0012	30	F	V	I	0.83	0.89	0.93	0.06	0.10	0.66	0.68	0.73	0.03	0.07
CIDP-MRU-0013	46	F	V	Af	0.69	0.76	0.83	0.07	0.14	0.59	0.61	0.66	0.02	0.08
CIDP-MRU-0014	46	F	IV	I	0.38	0.47	0.58	0.08	0.19	0.29	0.39	0.44	0.10	0.15
CIDP-MRU-0015	42	F	IV	M	0.63	0.76	0.71	0.13	0.09	0.50	0.59	0.59	0.09	0.08
CIDP-MRU-0016	39	F	IV	I	0.64	0.75	0.85	0.11	0.21	0.49	0.55	0.69	0.06	0.19
CIDP-MRU-0018	43	F	V	I	0.78	0.66	0.75	-0.12	-0.03	0.63	0.55	0.62	-0.08	-0.01
CIDP-MRU-0019	43	F	II	C	0.41	0.58	0.58	0.17	0.17	0.32	0.44	0.44	0.12	0.12
CIDP-MRU-0020	51	F	IV	A	0.69	0.87	0.61	0.18	-0.08	0.52	0.68	0.49	0.17	-0.03
CIDP-MRU-0021	54	F	IV	M	0.51	0.59	0.65	0.09	0.15	0.41	0.45	0.54	0.04	0.13
CIDP-MRU-0022	53	F	III	C	0.43	0.54	0.56	0.11	0.13	0.35	0.43	0.42	0.07	0.06
CIDP-MRU-0023	51	F	III	A	0.58	0.60	0.51	0.02	-0.07	0.42	0.40	0.37	-0.02	-0.05
CIDP-MRU-0024	34	F	III	C	0.69	0.75	0.86	0.06	0.17	0.56	0.60	0.68	0.05	0.13
CIDP-MRU-0025	43	F	III	M	0.56	0.69	0.64	0.14	0.09	0.46	0.56	0.47	0.10	0.02
CIDP-MRU-0026	49	F	IV	M	0.60	0.70	0.68	0.10	0.08	0.48	0.55	0.50	0.07	0.02
CIDP-MRU-0029	57	F	IV	M	0.46	0.47	0.49	0.01	0.04	0.34	0.34	0.35	0.01	0.01
CIDP-MRU-0030	33	F	III	C	0.78	0.78	0.80	0.00	0.02	0.64	0.64	0.63	0.00	-0.01
CIDP-MRU-0031	41	F	III	C	0.57	0.68	0.63	0.11	0.06	0.41	0.52	0.48	0.11	0.07
CIDP-MRU-0032	48	F	III	M	0.54	0.58	0.48	0.04	-0.05	0.42	0.43	0.36	0.01	-0.06
CIDP-MRU-0033	43	F	III	C	0.55	0.67	0.57	0.13	0.03	0.42	0.55	0.46	0.13	0.04

CIDP-MRU-0034	42	F	IV	M	0.60	0.72	0.70	0.13	0.10	0.46	0.53	0.52	0.07	0.06
CIDP-MRU-0035	48	F	III	M	0.52	0.60	0.65	0.08	0.12	0.42	0.46	0.52	0.05	0.10
CIDP-MRU-0036	42	F	V	Af	0.79	0.86	0.88	0.07	0.09	0.55	0.67	0.68	0.12	0.12
CIDP-MRU-0038	52	F	V	M	0.56	0.58	0.66	0.02	0.11	0.43	0.46	0.52	0.04	0.10
CIDP-MRU-0039	59	F	V	Af	0.70	0.82	0.89	0.12	0.20	0.52	0.64	0.70	0.12	0.18
CIDP-MRU-0040	49	F	III	C	0.42	0.42	0.59	-0.01	0.17	0.34	0.32	0.46	-0.02	0.12
CIDP-MRU-0041	34	F	IV	I	0.82	0.89	0.91	0.07	0.10	0.64	0.69	0.66	0.05	0.02
CIDP-MRU-0042	41	F	III	C	0.62	0.69	0.61	0.07	-0.01	0.46	0.56	0.48	0.10	0.02
CIDP-MRU-0044	55	F	IV	M	0.63	0.65	0.62	0.03	-0.01	0.53	0.51	0.47	-0.02	-0.06
CIDP-MRU-0045	57	F	IV	M	0.54	0.55	0.51	0.01	-0.03	0.39	0.45	0.36	0.05	-0.04
CIDP-MRU-0046	42	F	V	M	0.63	0.84	0.87	0.22	0.24	0.53	0.63	0.65	0.11	0.12
CIDP-MRU-0047	40	F	IV	M	0.62	0.62	0.75	0.00	0.13	0.48	0.48	0.55	0.00	0.06
CIDP-MRU-0048	45	F	IV	M	0.52	0.56	0.52	0.04	0.00	0.39	0.38	0.35	-0.01	-0.04
(CIDP-MRU-0017)	*-45*	*(F)**	*(III)**	*(C)**	*(0.56)**	*(DO)**	*(DO)**	*(DO)**	*(DO)**	*(0.45)**	*(DO)**	*(DO)**	*(DO)**	*(DO)**
*	*				*(0.40)**	*(DO)**	*(DO)**	*(DO)**	*(DO)**	*(0.32)**	*(DO)**	*(DO)**	*(DO)**	*(DO)**
(CIDP-MRU-0043)	*-44*	*(F)**	*(V)**	*(I)**	*(0.29)**	*(DO)**	*(DO)**	*(DO)**	*(DO)**	*(0.25)**	*(DO)**	*(DO)**	*(DO)**	*(DO)**
*	*				*(0.80)**	*(DO)**	*(DO)**	*(DO)**	*(DO)**	*(0.65)**	*(DO)**	*(DO)**	*(DO)**	*(DO)**
